# Infiltrating characteristics and prognostic value of tertiary lymphoid structures in resected gastric neuroendocrine neoplasm patients

**DOI:** 10.1002/cti2.1489

**Published:** 2024-02-05

**Authors:** Daming Cai, Xingzhou Wang, Heng Yu, Chunhua Bai, Yonghuan Mao, Mengjie Liang, Xuefeng Xia, Song Liu, Meng Wang, Xiaofeng Lu, Junfeng Du, Xiaofei Shen, Wenxian Guan

**Affiliations:** ^1^ Department of General Surgery, Nanjing Drum Tower Hospital, Affiliated Hospital of Medical School Nanjing University Nanjing China; ^2^ Dermatology and Interventional Surgery, Nanjing Drum Tower Hospital, Affiliated Hospital of Medical School Nanjing University Nanjing China; ^3^ Department of General Surgery, The 7th Medical Center Chinese PLA General Hospital Beijing China; ^4^ Department of General Surgery Drum Tower Clinical Medical College of Nanjing Medical University Nanjing China

**Keywords:** gastric neuroendocrine neoplasms, nomogram, prognosis, tertiary lymphoid structures, tumor immune microenvironment

## Abstract

**Objectives:**

Tertiary lymphoid structures (TLSs) are lymphocyte aggregates that play an anti‐tumor role in most solid tumors. However, the functions of TLS in gastric neuroendocrine neoplasms (GNENs) remain unknown. This study aimed to determine the characteristics and prognostic values of TLS in resected GNEN patients.

**Methods:**

Haematoxylin–eosin, immunohistochemistry (IHC) and multiple fluorescent IHC staining were used to assess TLS to investigate the correlation between TLSs and clinicopathological characteristics and its prognostic value.

**Results:**

Tertiary lymphoid structures were identified in 84.3% of patients with GNEN. They were located in the stromal area or outside the tumor tissue and mainly composed of B and T cells. A high density of TLSs promoted an anti‐tumor immune response in GNEN. CD15^+^ TANs and FOXP3^+^ Tregs in TLSs inhibited the formation of TLSs. High TLS density was significantly associated with prolonged recurrence‐free survival (RFS) and overall survival (OS) of GNENs. Univariate and multivariate Cox regression analyses revealed that TLS density, tumor size, tumor–node–metastasis (TNM) stage and World Health Organisation (WHO) classification were independent prognostic factors for OS, whereas TLS density, tumor size and TNM stage were independent prognostic factors for RFS. Finally, OS and RFS nomograms were developed and validated, which were superior to the WHO classification and the TNM stage.

**Conclusion:**

Tertiary lymphoid structures were mainly located in the stromal area or outside the tumor area, and high TLS density was significantly associated with the good prognosis of patients with GNEN. Incorporating TLS density into a nomogram may improve survival prediction in patients with resected GNEN.

## Graphical abstract

In this study, we found that tertiary lymphoid structures (TLSs) are anti‐tumor element of tumor immune microenvironment in patients with resected gastric neuroendocrine neoplasm (GNEN). High infiltration of TLSs is an independent and favourable prognosis predictor for GNENs. Our findings indicate that nomograms including TLS density can predict prognosis of GNEN patients more precisely.

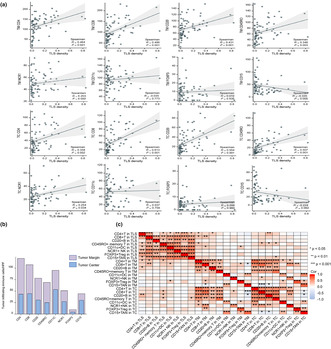

## Introduction

Gastric neuroendocrine neoplasm (GNEN) is a rare tumor derived from gastric neuroendocrine cells. According to the Surveillance, Epidemiology and End Results (SEER) database, the incidence of GNEN increased to 4.85/1 000 000 in 2014.^1^ The number of patients with GNEN has increased almost 15‐fold in the past 40 years.^2^ According to Japan's National Cancer Registry, the incidence of gastroenteropancreatic neuroendocrine neoplasms (GEP‐NENs) was 3.532/100 000 in 2016, with the most common primary site being the rectum (53%), followed by the pancreas (20%) and stomach (13%).^3^ The incidence of neuroendocrine neoplasms (NENs) in China was 1.14/100 000,^1^ and the incidence of GNEN was 2.1/1 000 000.^4^ According to epidemiological data of GEP‐NENs collected from 23 centres in China from 2000 to 2010, the percentage of GNEN was nearly 27% in all GEP‐NEN.^5^ The overall 5‐year relative survival of GNEN in China (29.1%) was lower than that in the United States (67.3%).^4^ At present, there are two traditional predictors to evaluate the prognosis of GNEN patients in clinical practice: the 8th edition of gastroenteropancreatic neuroendocrine tumor staging of the American Joint Committee on Cancer (AJCC8) and the 2019 World Health Organisation (WHO) classification of GEP‐NENs.^6^, ^7^ Currently, studies combining tumor–node–metastasis (TNM) stages, WHO classifications and immune factors for the prognostic prediction of recurrence‐free survival (RFS) and (or) overall survival (OS) of patients with GNEN are scarce.^8^, ^9^, ^10^ However, immune factors may play an important role in GNEN.^11^, ^12^, ^13^ Thus, a comprehensive understanding of GNEN and combining the findings of GNEN immunity are urgently needed.

Emerging evidence has shown that tertiary lymphoid structures (TLSs) play a crucial role in the tumor immune microenvironment (TIME).^14^, ^15^, ^16^ TLSs are ectopic lymphoid organs found in non‐lymphoid tissues at sites of chronic inflammation, including tumors. TLS is a protective predictor of prognosis in tumors such as breast cancer,^17^ lung cancer,^18^, ^19^ colorectal cancer,^20^ malignant melanoma,^21^ ovarian cancer,^22^ gastric cancer,^23^ pancreatic cancer,^24^ and oral squamous cell carcinoma.^25^ TLS is also associated with a favorable prognosis in most gastrointestinal tumors.^26^, ^27^, ^28^, ^29^, ^30^, ^31^ In addition, TLS might serve as a compartment for generating memory T and B cells, enhancing the anti‐tumor immune response.^32^ Although most studies reported that the presence of TLS is associated with good patient survival,^17^, ^18^, ^19^, ^20^, ^21^, ^22^, ^23^, ^24^, ^25^ Finkin *et al*.^33^ concluded that the presence of TLS was a poor prognostic factor for hepatocellular carcinoma (HCC). Although existing evidence has shown that TLSs may have an impact on tumor development, the prognostic role of TLSs in NENs has not been extensively investigated. It has been shown that TLS can participate in the development of pancreatic neuroendocrine tumors (NETs) and small bowel NETs^34^, ^35^; however, the functions and prognostic value of TLSs in GNEN remain elusive. Therefore, the present multicentre retrospective cohort study endeavoured to explore the prognostic role and functions of TLSs in patients with resected GNEN.

## Results

### Clinicopathological characteristics

The median follow‐up duration of the training set was 42 months (range, 1–95 months). The median follow‐up period for the external validation set was 51 months (range, 2–95 months). The clinicopathological characteristics of the two patient cohorts are summarised in Table 1. In the training cohort, more than half of the patients were men (58.6%), and the median age was 62.5 years (interquartile range [IQR], 51–69.25 years). The percentages of patients with AJCC8 TNM stages I, II, III and IV were 46.4%, 10.7%, 35.0% and 7.9%, respectively, and those with WHO grades 1, 2 and 3 and neuroendocrine carcinoma (NEC)/mixed neuroendocrine–non‐neuroendocrine neoplasms (MINEN) were 37.9%, 10.7% and 51.4% respectively. More than half of the tumors in the patients with resected GNEN were less than 5 cm in diameter (69.3%). The median of TLS density was 0.07 (IQR, 0.02–0.2 count mm^−2^). These metrics were not significantly different from those in the external validation cohort.

**Table 1:** Demographics and clinical characteristics of the training cohort and external validation set of GNEN

Characteristics	Training set (*n* = 140)	External validation set (*n* = 126)	*P*‐value
Age, median (IQR)	62.5 (51, 69.25)	63 (51.25, 68)	0.912
Gender, *n* (%)			
Female	58 (41.4%)	43 (34.1%)	0.272
Male	82 (58.6%)	83 (65.9%)	
WHO classification, *n* (%)			
NET G1	53 (37.9%)	40 (31.7%)	0.479
NET G2/ G3	15 (10.7%)	18 (14.3%)	
NEC + MiNEN	72 (51.4%)	68 (54.0%)	
T stage, *n* (%)			
T1	62 (44.3%)	49 (38.9%)	0.348
T2	13 (9.3%)	19 (15.1%)	
T3	54 (38.6%)	44 (34.9%)	
T4	11 (7.8%)	14 (11.1%)	
N stage, *n* (%)			
N0	79 (56.4%)	80 (63.5%)	0.295
N+	61 (43.6%)	46 (36.5%)	
M stage, *n* (%)			
M0	135 (96.4%)	117 (92.9%)	0.304
M1	5 (3.6%)	9 (7.1%)	
AJCC 8th TNM stage, *n* (%)			
I	65 (46.4%)	58 (46.1%)	0.971
II	15 (10.7%)	12 (9.5%)	
III	49 (35.0%)	47 (37.3%)	
IV	11 (7.9%)	9 (7.1%)	
Tumor size, *n* (%)			
≥ 5 cm	43 (30.7%)	36 (28.6%)	0.805
< 5 cm	97 (69.3%)	90 (71.4%)	
TLS density, median (IQR)	0.07 (0.02, 0.2)	0.13 (0.05, 0.2)	0.091

GNEN, gastric neuroendocrine neoplasms; IQR, Interquartile range; N0, Negative lymph node metastasis. N+, Positive lymph node metastasis; TLS, tertiary lymphoid structures; TNM, tumor–node–metastasis.

### Characteristics of TLSs in different WHO classifications and TNM stages

**Figure 1:**
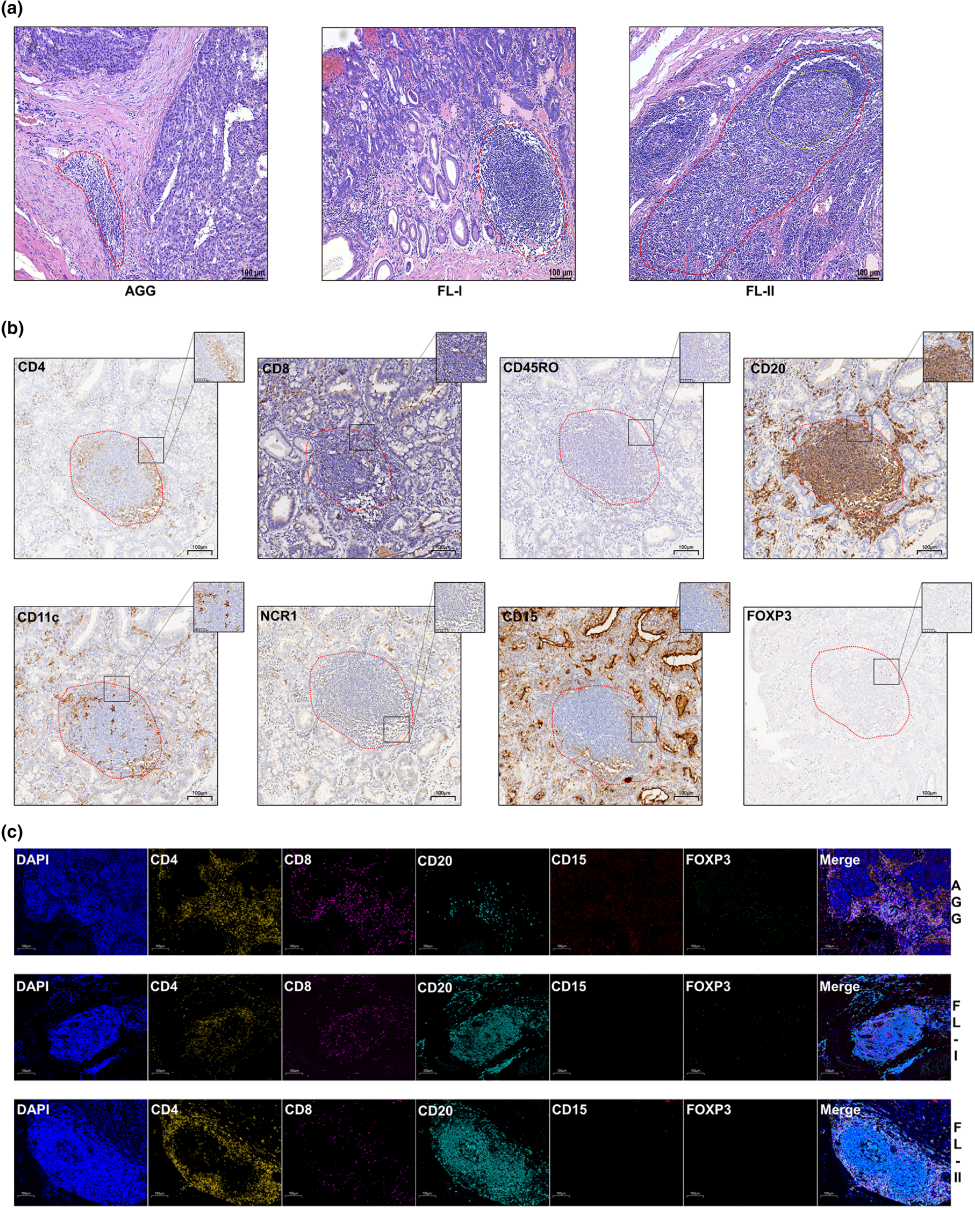
H&E staining, immunohistochemistry and multispectral fluorescent immunohistochemistry of tertiary lymphoid structures (TLS). **(a)** Representative H&E‐staining images for AGG, FL‐I and FL‐II (the red dotted line represents TLS region and the yellow dotted line represents the germinal centre in TLS). **(b)** Immunohistochemistry images showing the cellular compositions of TLS: CD4^+^ T cells; CD8^+^ T cells; CD45RO^+^memory T cells; CD20^+^ B cells; CD11c^+^DC cells; NCR1^+^ NK cells; CD15^+^ TANs; and FOXP3^+^ Tregs. **(c)** Multispectral fluorescent immunohistochemistry images of TLS displaying the cellular compositions and mature forms of TLS intuitively. Magnification: 200×. Scale bars correspond to 100 μm. (TLS maturity classification: AGG/FL‐I/FL‐II. Aggregates (AGG) are almost squashed, elongated or teardrop shaped; primary mature TLS (FL‐I) appears round or oval; and secondary mature TLS (FL‐II) contains a germinal centre).

**Figure 2:**
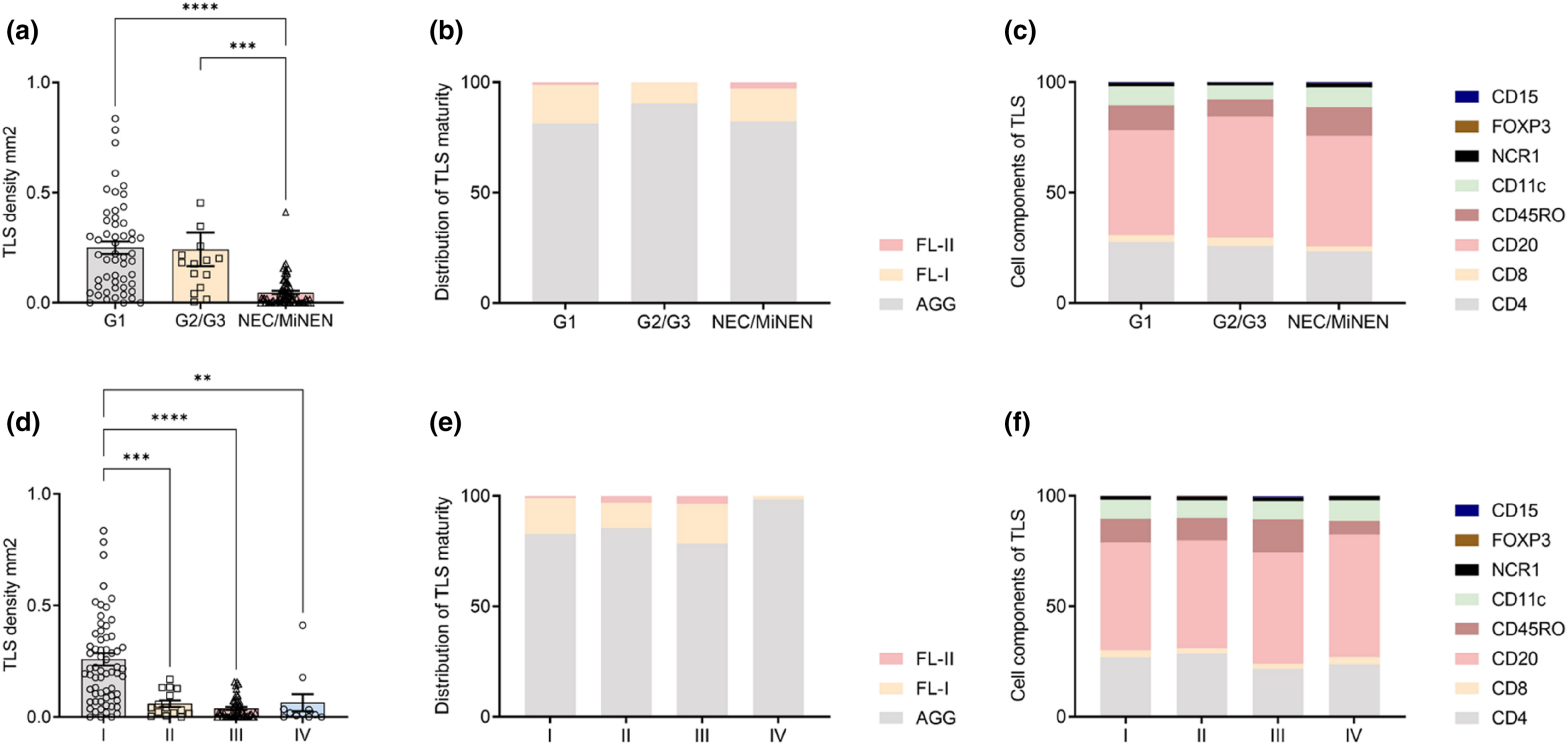
**(a)** The relationship between the tertiary lymphoid structures (TLS) density and WHO classification. (The WHO classifications contain G1, G2/G3 and NEC/MiNEN.) **(b)** The association of the distribution of TLS maturity with WHO classification (the TLS maturity is described in the main text). **(c)** The association of the cell components of TLS with WHO classification (the cell components of TLS are described in the main text). **(d)** Association of the TLS density with the AJCC 8th TNM grade. (The AJCC 8th TNM grade includes I, II, III and IV.) **(e)** The association of the distribution of TLS maturity with AJCC 8th TNM grade. **(f)** Relationship between the cell components of TLS and AJCC 8th TNM grade. ** *P* < 0. 01, *** *P* < 0.001 and **** *P* < 0.0001.

The density, location, maturity and cellular composition of TLSs were detected in all whole‐slide images (WSIs). To quantify the density of TLSs, TLS negative was indicated by a TLS density of 0. TLS was mainly located at the margin of the tumor tissue or in the peritumorally stroma. TLS was not observed in the tumor parenchyma. TLSs occurred in various sizes and shapes and were divided into three forms of maturity as follows. Aggregates (AGG), which are primary TLSs, were almost squashed, elongated, or teardrop shaped; primary follicles (FL‐I), which are primary mature TLSs, were usually round or oval; and secondary follicles (FL‐II), which are secondary mature TLSs containing a germinal centre (GC), were usually large and round (Figure 1a and c). The three mature forms of TLSs occurred in almost every WHO classification and TNM stage. For the cellular composition of TLSs, CD4^+^ T cells and CD8^+^ T cells were mainly distributed in the peripheral region of the TLSs, and CD4^+^ T cells infiltrated more than CD8^+^ T cells on average. CD20^+^ B cells were primarily located in the centre of the TLSs, whereas CD45RO+ memory T cells and CD11c^+^ DCs were distributed primarily within the T‐cell zone, with some dispersion in the centre of the TLSs. Only sporadic CD15^+^ TANs and NCR1^+^ NK cells were detected. FOXP3^+^ Tregs were rarely observed in the TLS zone (Figure 1b). TLS densities of G1 and G2/G3 were significantly higher than those of NEC/MiNEN; however, no significant difference was found between G1 and G2 (Figure 2a). The densities of AGG and FL‐I were significantly higher in G1 than in NEC/MiNEN; however, there was no statistically significant difference in the FL‐II density (Supplementary figure 1a and b). The distribution of TLS maturity was similar under the different WHO classifications (Figure 2b; Supplementary figure 2a). The proportion of the primary cell components of TLS among different WHO classifications did not show significant differences; only the proportion of FOXP3^+^ Tregs and CD15^+^ TANs were higher in NEC/MiNEN than in G1 (Figure 2c; Supplementary figure 2b). The TLS density of the TNM I stage was significantly higher than that of the TNM II, III and IV stages (Figure 2d). AGG and FL‐I densities of the TMN I stage were significantly higher than those of the TNM III stage; however, the FL‐II density did not differ among different TNM stages (Supplementary figure 3a and b). The distribution of the TLS maturity was similar for the same TNM stage (Figure 2e; Supplementary figure 4a). In addition to FOXP3^+^ Tregs and CD15^+^ TANs in the TLSs, there were no differences in the proportion of primary cell components of TLS with different TNM stages (Figure 2f; Supplementary figure 4b). Collectively, these data suggest that a higher density of TLS, rather than TLS maturity and cell compositions, is linked to favorable grading and staging outcomes for GNEN.

### Relationship between TLS density and tumor‐infiltrating immune cells outside the TLS zone located at the tumor margin or tumor centre

**Figure 3:**
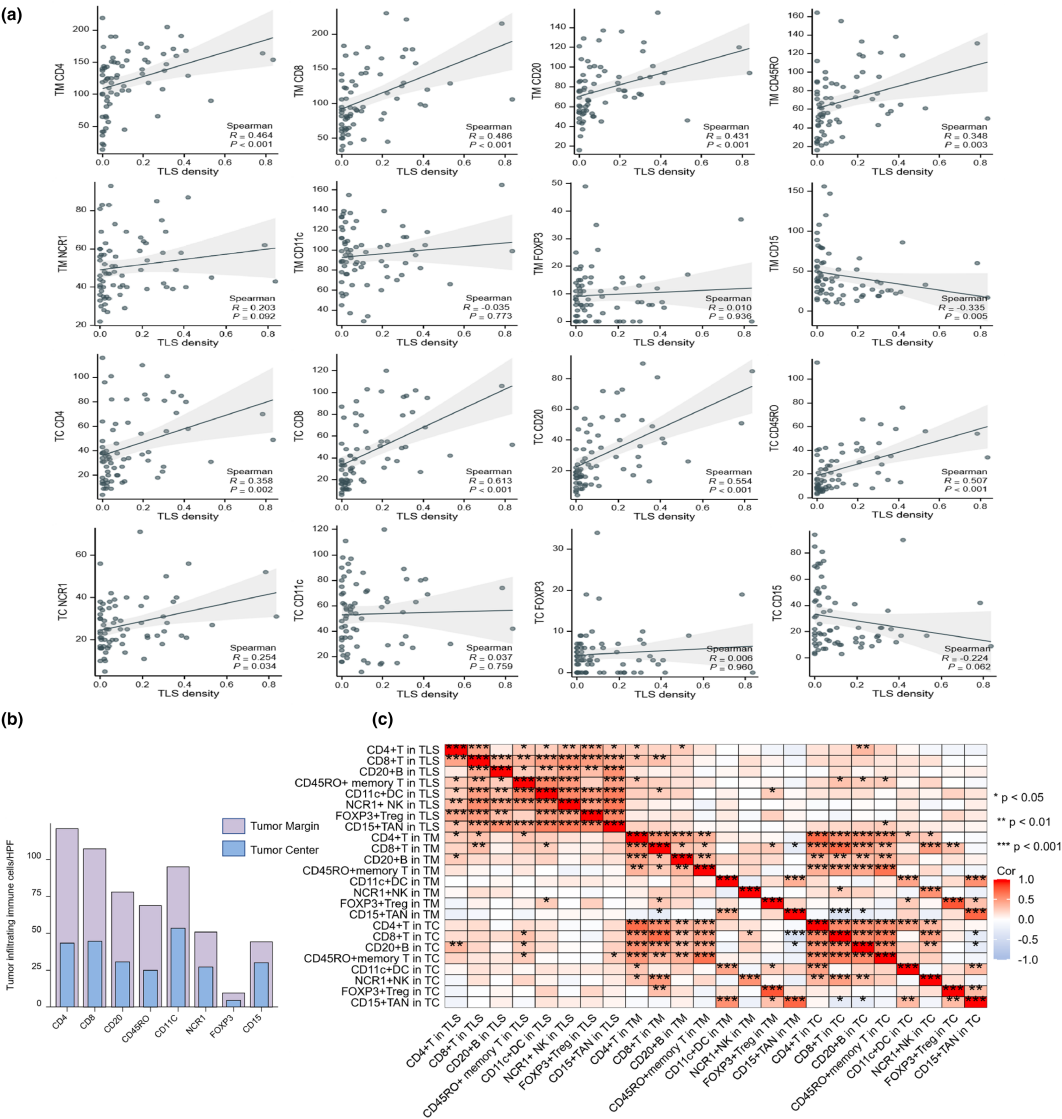
**(a)** Correlation between tumor‐infiltrating immune cells located at the tumor margin or tumor centre outside the tertiary lymphoid structures (TLS) zone and TLS density. (TM refers to tumor margin and TC means tumor centre). **(b)** The components and infiltration profile of main immune‐infiltrating cells located at the tumor areas outside TLS zone of gastric neuroendocrine neoplasms. (The purple bar indicates the tumor‐infiltrating immune cells located at the tumor margin outside TLS zone every high‐power field (HPF); and the blue bar indicates the tumor‐infiltrating immune cells located at the tumor centre outside TLS zone every HPF.) **(c)** The heatmap image showing the correlation of the tumor‐infiltrating immune cells in the TLS zone, the tumor‐infiltrating immune cells located at the tumor margin outside the TLS zone and the tumor‐infiltrating immune cells located at the tumor centre outside the TLS zone.

Based on the identification of TLSs in GNEN tissues, we further explored the relationship between the TLS density and tumor‐infiltrating immune cells located at the tumor margin or tumor centre. The results showed a significant positive correlation between TLS density and tumor‐infiltrating CD4^+^ T cells, CD8^+^ T cells, CD20^+^ B cells and CD45RO^+^ memory T cells (Spearman coefficient (*ρ*) = 0.464, 0.486, 0.431 and 0.348; *P* < 0.001, < 0.001, < 0.001 and = 0.003, respectively). Meanwhile, CD15^+^ TANs were negatively correlated with the density of TLS (*ρ* = −0.335; *P* = 0.005). NCR1^+^ NK cells were positively correlated with TLS density but with no significant difference (*P =* 0.092). No significant correlation was found between TLS density and CD11c^+^ DCs and FOXP3^+^ Tregs. Similarly, CD4^+^ T cells, CD8^+^ T cells, CD20^+^ B cells, CD45RO^+^ memory T cells and NCR1^+^ NK cells located in the tumor centre outside the TLS were significantly positively correlated with the density of TLSs in GNEN (*ρ* = 0.358, 0.613, 0.554, 0.507 and 0.254; *P* = 0.002, < 0.001, < 0.001, < 0.001 and = 0.034, respectively). In contrast, CD15^+^ TANs were negatively correlated with the density of TLS but with no significant difference (ρ = −0.224; *P* = 0.062). No significant correlation was found between the density of TLS and CD11c^+^ DCs and FOXP3^+^ Tregs (Figure 3a). Together, these data suggest that a high TLS density may promote an immune‐responsive microenvironment.

### Relationship between TLS cell components and tumor‐infiltrating immune cells outside the TLSs located at the tumor margin or tumor centre

The number of tumor‐infiltrating immune cells outside the TLSs located at the tumor margin was greater than that at the tumor centre, and the main tumor‐infiltrating immune cells were CD4^+^ T cells, CD8^+^ T cells, CD20^+^ B cells, CD45RO^+^ memory T cells, CD11c^+^ DCs, NCR1^+^ NK cells, FOXP3^+^ Tregs and CD15^+^ TANs (Figure 3b). Next, the correlations between various cellular components within the TLS and their relationships with infiltrating immune cells outside the TLSs located at the tumor margin or tumor centre were analysed using heatmaps. The results revealed that cells in the TLSs were positively correlated with each other, except for CD4^+^ T cells and CD20^+^ B cells (ρ = 0.1, *P* = 0.4), as well as CD45RO^+^ memory T cells and FOXP3^+^ Tregs (ρ = 0.22, *P* = 0.06). Meanwhile, CD4^+^ T cells and CD8^+^ T cells located at the tumor margin outside the TLSs were positively correlated with CD4^+^ T cells and CD8^+^ T cells in the TLS (ρ = 0.24, 0.31, *P* = 0.04, < 0. 01, respectively). In addition, CD45RO^+^ memory T cells located at the tumor centre outside the TLSs were positively correlated with the CD45RO^+^ memory T cells in the TLSs (ρ = 0.25, *P* = 0.04; Figure 3c). Overall, these data indicate that the crosstalk between TLS and other immune cells upregulates the anti‐tumor immune response.

### Characteristics of TLSs in patients with resected GNEN based on multiple fluorescent IHC (mIHC) staining

**Figure 4:**
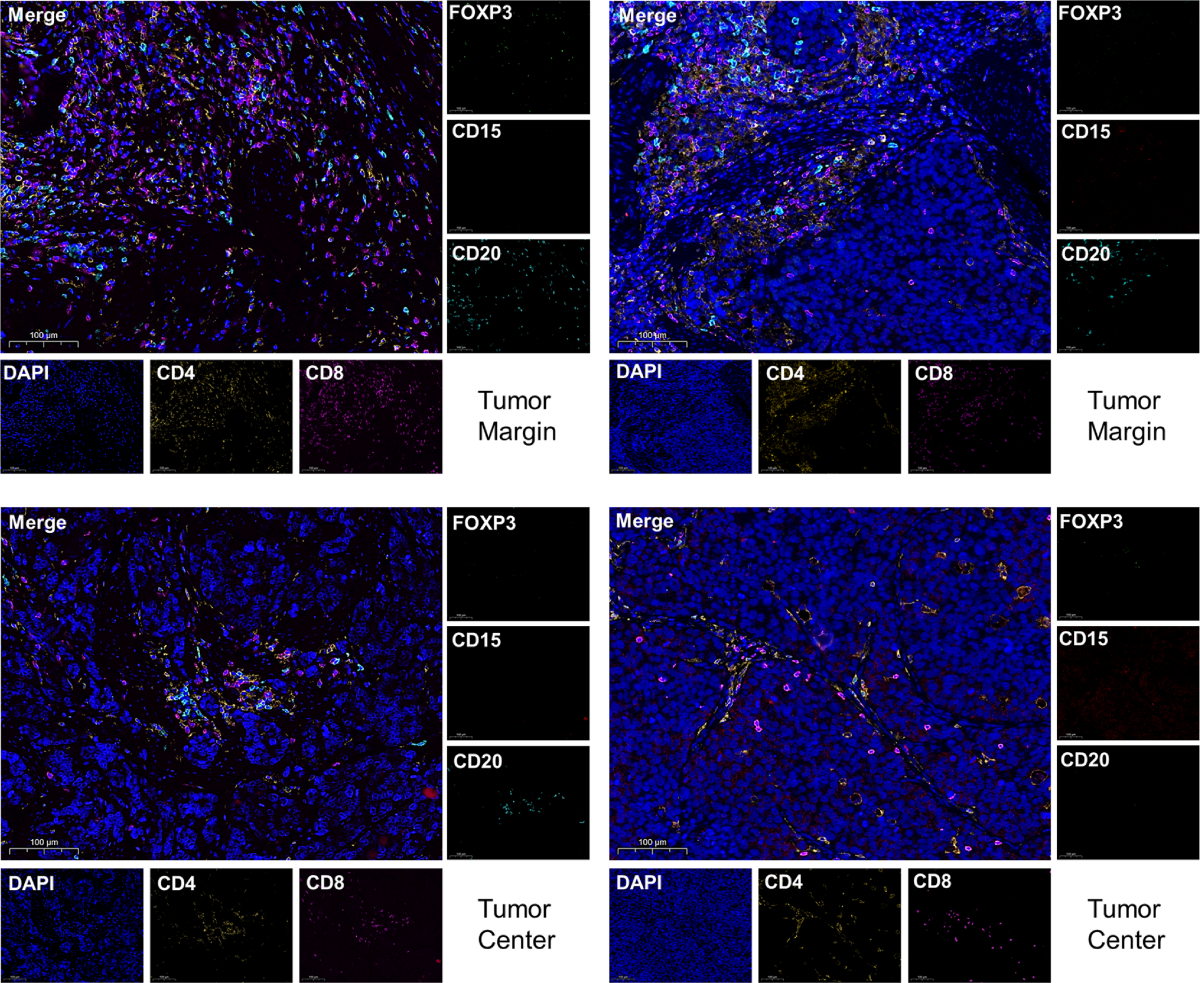
Multispectral fluorescent immunohistochemistry of tumor‐infiltrating immune cells outside the tertiary lymphoid structures (TLS) located at the tumor margin or tumor centre in gastric neuroendocrine neoplasms (GNENs) patients with high TLS density and low TLS density. Tumor margin: from left to right, it presents the representative immunofluorescence images of tumor‐infiltrating immune cells outside the TLS located at the tumor margin in GNEN patients with high TLS density and low TLS density. Tumor centre: from left to right, it presents representative immunofluorescence images of tumor‐infiltrating immune cells outside the TLS located at the tumor centre in GNEN patients with high TLS density and low TLS density. Magnification: 400×. Scale bars correspond to 100 μm.

CD4^+^ T cells, CD8^+^ T cells, CD20^+^ B cells, CD15^+^ TANs and FOXP3^+^ Treg were selected based on the results of IHC staining to simultaneously evaluate the localisation and abundance of multiple immune cells of TLS. mIHC‐staining results revealed that CD4^+^ T cells and CD8^+^ T cells were mainly distributed in the peripheral region of TLSs, with greater infiltration of CD4^+^ T cells than CD8^+^ T cells. Moreover, CD20^+^ B cells were primarily located in the centre of the TLSs. mIHC staining revealed that the proportions of TANs and TLS Tregs were significantly higher in GNEC/MiNEN than in G1 (Supplementary Figure 5a and b). These results were consistent with those of IHC staining. This might be a unique feature of TLSs in GNEN that is different from that in other tumor types. Meanwhile, mIHC staining showed that tumor‐infiltrating CD4^+^ T cells, CD8^+^ T cells and CD20^+^ B cells outside the TLS zone were higher in patients with a high TLS density than in those with a low TLS density; however, the infiltration of CD15^+^ TANs was low (Figure 4). Taken together, mIHC‐staining data more intuitively suggested that a high density of TLS might play an anti‐tumor role in the TME of GNEN.

### Association of TLS density with the survival of GNEN patients

**Figure 5:**
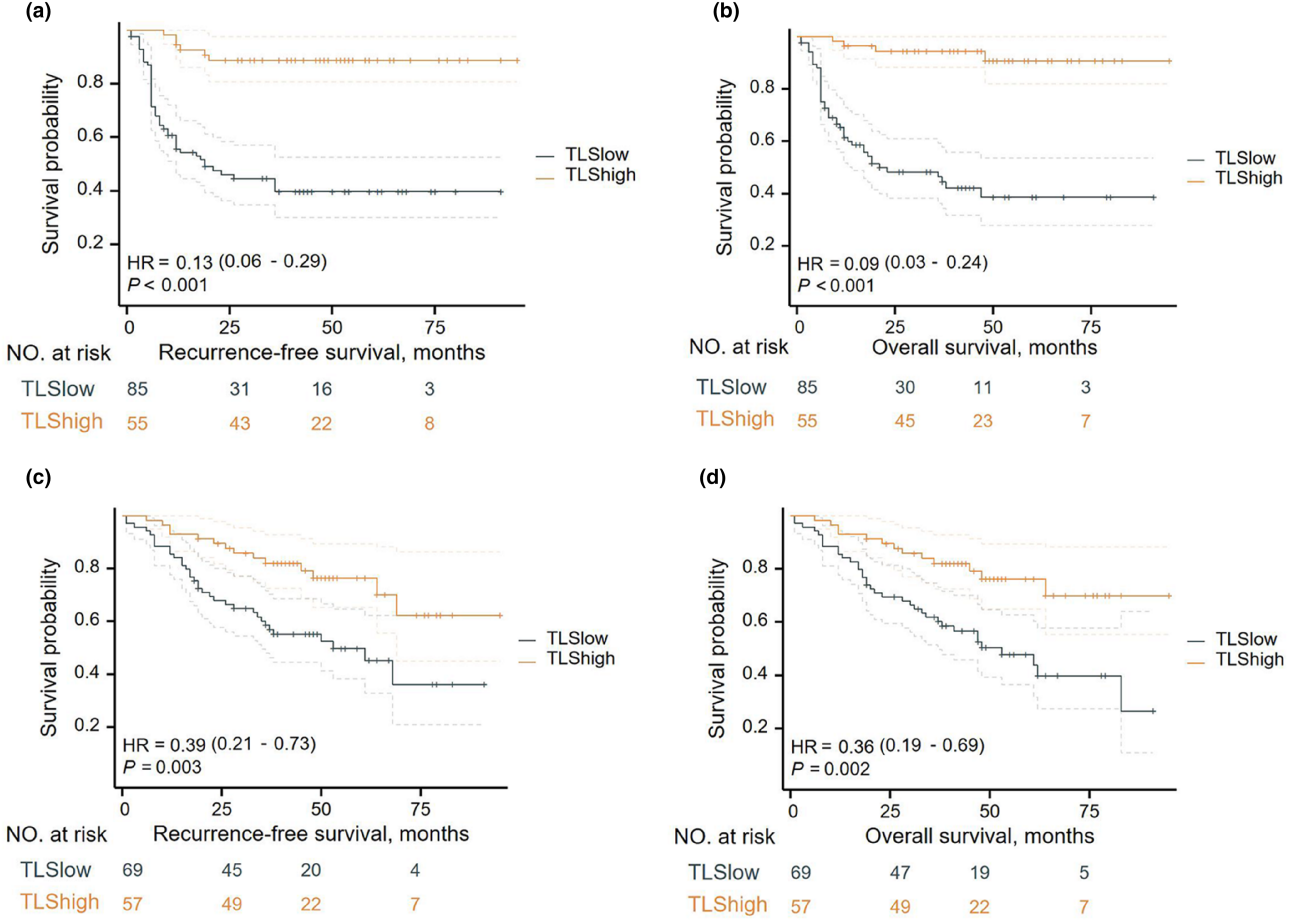
Kaplan–Meier survival analyses for recurrence‐free survival (RFS) based on the density of tertiary lymphoid structures (TLS) in the training cohort **(a)**. Kaplan–Meier survival analyses for the overall survival (OS) based on the density of TLS in the training cohort **(b)**. Kaplan–Meier survival analyses for RFS and OS were conducted based on the density of TLS in the external validation cohort **(c, d)**.

Next, receiver operating characteristic (ROC) curves were drawn and the best cut‐off value for TLS density was determined. The best cut‐off value for TLS density was 0.1 with an area under the curve (AUC) value of 0.799 (95% confidence interval (CI) 0.726–0.872, Supplementary figure 6). The included population was divided into TLS^High^ and TLS^Low^ groups based on the cut‐off value. The TLS density was significantly associated with the prolonged RFS in resected GNEN patients (hazard ratio [HR] = 0.13 95% CI 0.06–0.29, *P* < 0.001, Figure 5a). The TLS density was significantly associated with the prolonged OS in resected GNEN patients (HR = 0.09, 95% CI 0.03–0.24, *P* < 0.001, Figure 5b). Based on the optimal cut‐off value for TLS density, it was found that a high TLS density was significantly associated with prolonged RFS and OS in resected GNEN patients in the external validation set **(**Figure 5c and d). Univariate and multivariate survival analyses revealed that TLS density, TNM stage and tumor size were independent prognostic factors for RFS (HR = 0.417, 95% CI 0.176–0.992, *P* = 0.048; HR = 1.856, 95% CI 1.030–3.344, *P* = 0.04; HR = 1.780, 95% CI 1.159–2.736, *P* = 0.009, respectively), whereas TLS density, tumor size, TNM stage and WHO classification were independent prognostic factors for OS in the training cohort (HR = 0.436, 95% CI 0.199–0.957, *P* = 0.038; HR = 2.394, 95% CI 1.2814–4.475, *P* = 0.006; HR = 1.597, 95% CI 1.040–2.453, *P* = 0.032; and HR = 2.074, 95%CI 1.014–4.241, *P* = 0.046, respectively). These results were validated using the external validation set (Table 2).

**Table 2:** Univariate and multivariate Cox regression analysis for RFS and OS of patients with GNEN. (Training set and external validation set)

	Training cohort (*n* = 140)				External validation set (*n* = 126)			
	Univariate analysis		Multivariate analysis		Univariate analysis		Multivariate analysis	
	Hazard ratio (95% CI)	*P*‐value	Hazard ratio (95% CI)	*P*‐value	Hazard ratio (95% CI)	*P*‐value	Hazard ratio (95% CI)	*P*‐value
RFS								
Age	1.850 (1.095–3.125)	**0.021**	1.176 (0.685–2.018)	0.556	1.183 (0.652–2.147)	0.579	–	–
TLS density	0.130 (0.059–0.286)	**< 0.001**	0.417 (0.176–0.992)	**0.048**	0.392 (0.210–0.732)	**0.003**	0.474 (0.245–0.916)	**0.026**
Tumor size	5.639 (3.321–9.576)	**< 0.001**	1.856 (1.030–3.344)	**0.040**	11.031 (5.250–23.175)	**< 0.001**	3.549 (1.504–8.374)	**0.004**
AJCC 8th TNM stage	2.908 (2.226–3.800)	**< 0.001**	1.780 (1.159–2.736)	**0.009**	3.168 (2.323–4.321)	**< 0.001**	2.101 (1.384–3.189)	**< 0.001**
WHO classification	4.971 (2.934–8.421)	**< 0.001**	1.572 (0.740–3.341)	0.239	3.930 (2.274–6.793)	**< 0.001**	1.571 (0.831–2.970)	0.164
Vascular invasion	7.282 (4.073–13.019)	**< 0.001**	1.117 (0.522–2.390)	0.776	2.464 (1.371–4.426)	**0.003**	0.665 (0.330–1.340)	0.253
Perineural invasion	5.714 (3.358–9.721)	**< 0.001**	1.144 (0.604–2.165)	0.680	2.688 (1.511–4.780)	**< 0.001**	1.059 (0.544–2.061)	0.866
OS								
Age	1.337 (0.809–2.208)	0.257	–	–	1.087 (0.605–1.953)	0.780	–	–
TLS density	0.09 (0.03–0.24)	**< 0.001**	0.436 (0.199–0.957)	**0.038**	0.365 (0.193–0.689)	**0.002**	0.477 (0.249–0.917)	**0.026**
Tumor size	7.554 (4.391–12.994)	**< 0.001**	2.394 (1.281–4.475)	**0.006**	8.733 (4.328–17.622)	**< 0.001**	2.662 (1.207–5.873)	**0.015**
AJCC 8th TNM stage	2.994 (2.295–3.906)	**< 0.001**	1.597 (1.040–2.453)	**0.032**	2.949 (2.175–3.999)	**< 0.001**	1.806 (1.207–2.702)	**0.004**
WHO classification	5.437 (3.242–9.120)	**< 0.001**	2.074 (1.014–4.241)	**0.046**	4.998 (2.621–9.533)	**< 0.001**	2.340 (1.145–4.780)	**0.020**
Vascular invasion	8.273 (4.646–14.730)	**< 0.001**	1.257 (0.543–2.908)	0.594	2.245 (1.258–4.008)	**0.006**	0.561 (0.287–1.095)	0.090
Perineural invasion	6.038 (3.561–10.238)	**< 0.001**	0.951 (0.481–1.877)	0.884	2.708 (1.526–4.804)	**< 0.001**	1.304 (0.675–2.519)	0.430

GNEN, gastric neuroendocrine neoplasms; OS, overall survival; RFS, recurrence‐free survival; TLS, tertiary lymphoid structures; TNM, tumor–node–metastasis. Bold values indicate significant variables in the survival analysis of patients with gastric neuroendocrine neoplasm.

### Construction of nomograms for predicting the probability of 3‐ and 5‐year OS and RFS in resected GNEN patients

**Figure 6:**
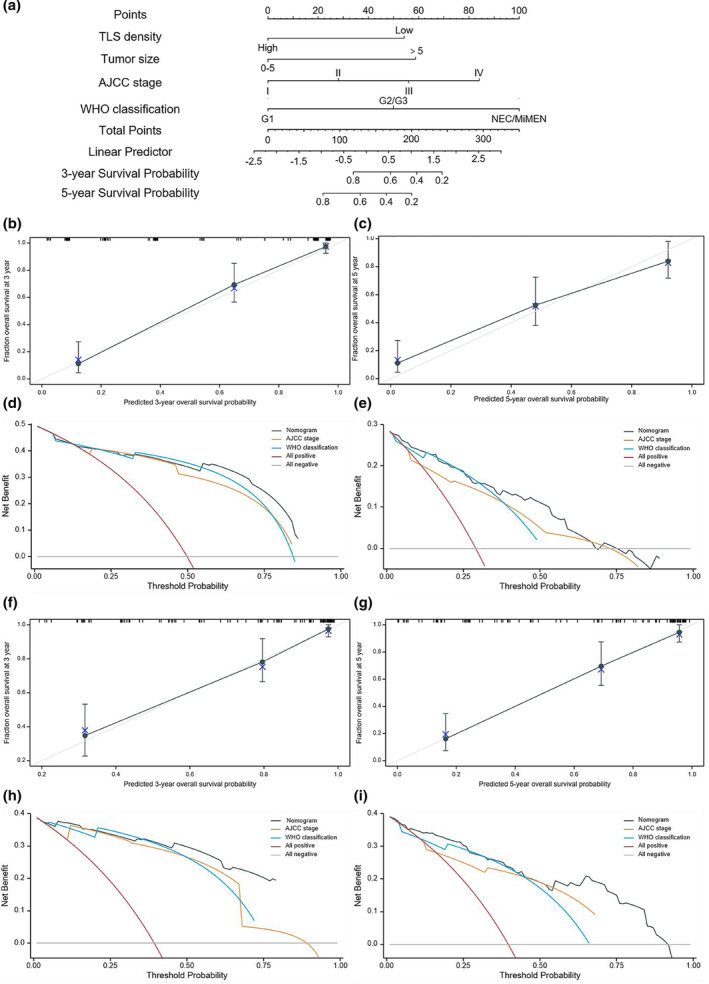
**(a)** A nomogram was constructed considering four factors: tertiary lymphoid structures (TLS) density, tumor size, AJCC 8th tumor–node–metastasis (TNM) stage and WHO classification to predict the probability of OS at 3 and 5 years. The probabilities were estimated as the sum of points for each variable as a function of total points. Each component was assigned points by tracing a line upwards from the corresponding values to the ‘point’ line. The total sum of points contributed by each variable was displayed on the ‘total points’ line. To determine the associated probability forecasts, a line was drawn downwards from the total points. The Bootstrap method was employed for internal validation, involving 1000 repeated samples. Calibration curves of the model in the training set **(b, c)** and decision curve analysis (DCA) of nomogram, AJCC 8th TNM grade and WHO classification for 3‐ and 5‐year OS in the training set **(d, e)**. Calibration curves of the model in the external validation set **(f, g)** and DCA curve of nomogram, AJCC 8th TNM grade and WHO classification for 3‐ and 5‐year OS in the external validation set **(h, i)**.

**Figure 7:**
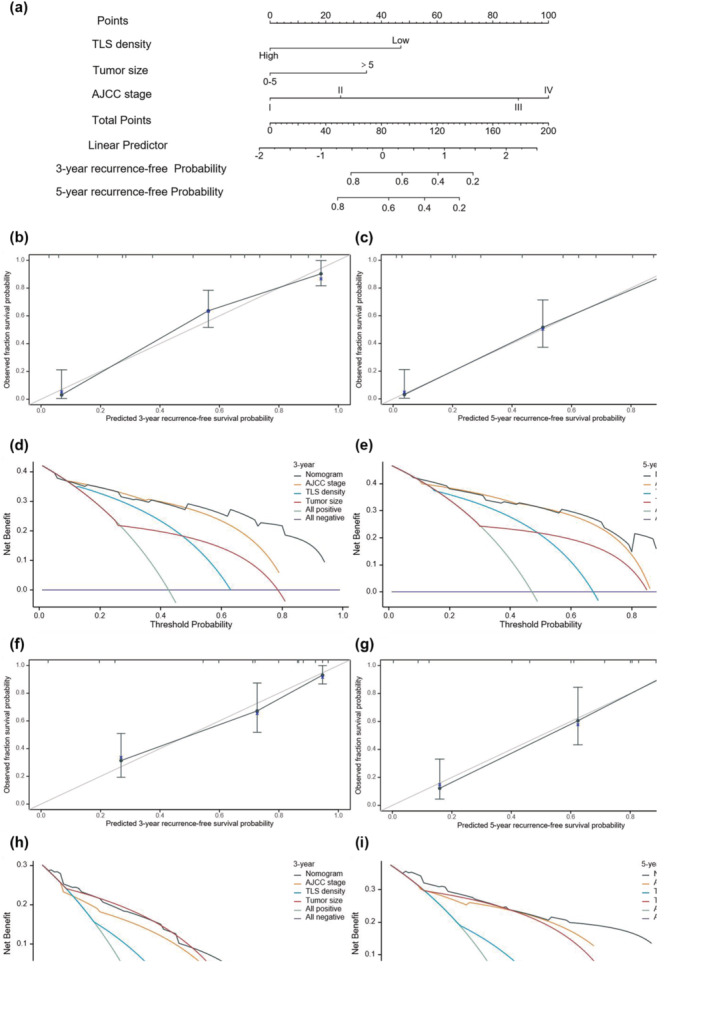
**(a)** A nomogram was constructed based on three factors: tertiary lymphoid structures (TLS) density, tumor size and AJCC 8th tumor–node–metastasis (TNM) stage for predicting the probability of RFS at 3 and 5 years. The probabilities were estimated as the sum of points for each variable as a function of total points. Each component was assigned points by drawing a line upwards from the matching values to the ‘point’ line. On the ‘total points’ line, the total sum of points added by each variable is shown. A line was drawn downward to read the associated probability forecasts. The Bootstrap method was used for internal validation, with 1000 repeat samples. Calibration curves for the model in the training set **(b, c)** and decision curve analysis (DCA) of the model in the training set (**d, e**). Calibration curves for the model in the external validation set **(f, g)** and DCA curve for the model in the external validation set **(h, i)**.

A nomogram for predicting 3‐ and 5‐year OS was built based on TLS density, tumor size, AJCC8 TNM stage and WHO classification (Figure 6a). The C‐index of this nomogram was 0.838 (0.819–0.857) in the training cohort and 0.848 (0.824–0.871) in the external validation set, which showed excellent predictive power. In addition, the C‐index of the nomogram was higher than that of the WHO classification (0.79), AJCC8 TNM stage (0.807), TLS density (0.698) and tumor size (0.721) in the training cohort. The C‐index of the nomogram was also higher than that of the WHO classification (0.745) and AJCC8 TNM stage (0. 776), TLS density (0.617) and tumor size (0.753) in the external validation set. Meanwhile, calibration curves showed that the nomogram had an excellent calibration ability both in the training set (Figure 6b and c) and in the external validation set (Figure 6f and g). Furthermore, when the threshold probability displayed at the *x*‐axis of the decision curve analysis (DCA) curves was about 0.5–0.75, the net benefit displayed at the *y*‐axis of the DCA curves revealed that our nomogram model significantly outperformed TNM staging and WHO classification in both the training set (Figure 6d and e) and the external validation set (Figure 6h and i), suggesting a great potential clinical application value of this nomogram. Moreover, the AUCs of time‐dependent ROC of GNEN patients for 3‐ and 5‐year OS prediction in the training and validation sets were 0.944/0.903, 0.732/0.756, 0.829/0.806, 0.218/0.184 and 0.798/0.737 and 0.878/0.863, 0.781/0.801, 0.778/0.773, 0.378/0.364 and 0.796/0.772, for the nomogram, AJCC8 TNM stage, WHO classification, TLS density and tumor size respectively (Supplementary figures 7 and 8). These data demonstrated that the discrimination of the nomogram was better than that of the four independent predictors. The nomogram for predicting 3‐ and 5‐year RFS was established based on included TLS density, tumor size and AJCC8 TNM stages (Figure 7a). The RFS model performed well in the training and validation sets (Figure 7b–i). The AUC of the corresponding time‐dependent ROC curve also showed superior performance (Supplementary figures 9 and 10). Both OS and RFS nomograms performed better than other predictors in GNET and GNEC patients in the training and validation sets (Supplementary figures 11 and 12).

## Discussion

The current study analysed two cohorts, consisting of 140 GNEN patients in the internal training set and 126 GNEN patients in the external validation set, to better understand the functional role of TLS in GNEN. The location, density, maturity, cellular components and prognostic value of TLS in GNEN were comprehensively analysed. It was found that TLS density was higher in G1 and G2/G3 than in NEC/MiNEN and TNM stage I than in TNM stages II, III and IV. A high TLS density was associated with good clinical results. The maturity and cellular components of TLS did not significantly differ among different WHO classifications and TNM stages. Predictive nomograms were constructed using the TLS density and other independent prognostic factors. After internal and external validation, the nomogram predicting the 3‐ and 5‐year OS and RFS of patients with resected GNEN performed well.

Tertiary lymphoid structures are ectopic and clustered lymphoid structures in chronic inflammatory tissues and the TIME.^15^ The present study characterised TLS density and found a good prognostic value of TLS density in GNEN. The proportion of TLSs was 84.3% of GNENs, which is similar to the presence of TLS in HCC (89%),^36^ lung squamous cell carcinoma (97%)^19^ and colorectal cancer (97%).^37^ Our data showed that a high TLS density was associated with prolonged survival, which is consistent with previous studies of pancreatic neuroendocrine tumors.^34^

Furthermore, the distribution of TLS was investigated at different maturity stages based on different WHO classifications and TNM stages of GNEN. It was found that AGG and FL‐I were common, while secondary mature TLS‐containing GCs (FL‐II) were rare, which is similar to the findings of Calderaro, in which the distribution of TLS maturity was AGG, FL‐I and FL‐II in 72 (56%), 43 (33%) and 14 (11%) among TLS^+^ HCC cases respectively.^36^ No differences in the distribution of TLS were found at different maturity stages based on different WHO classifications and TNM stages.

Cellular components and locations of TLS in the GNENs were further evaluated. As anticipated, the majority of cellular components in TLS were immune cells, including CD20^+^ B cells, CD45RO^+^ memory T cells, CD4^+^ T cells, CD8^+^ T cells, NCR1^+^ NK cells and CD11c^+^ DCs. We also identified immunosuppressive subsets, such as CD15^+^ TANs and FOXP3^+^ Tregs, but the proportion of these immunosuppressive subsets was low. TLS was mainly composed of B‐cell zones (some with GC) inside, T‐cell zones with DCs outside and NK cells, Tregs and CD15^+^ TANs were scattered in the TLSs, with a distribution similar to that of the secondary lymphatic organ. The cellular composition of TLS was also compared based on different WHO classifications and TNM stages. It was found that the main cellular components of TLS were similar in every WHO classification and TNM stage, except for CD15^+^ TANs and FOXP3^+^ Tregs, which inhibited the formation of TLS in line with the low TLS density in NEC/MiNEN. This might be a characteristic of TLS in GNEN that is different from that in other tumor types.

Correlations between TLS density and tumor‐infiltrating immune cells outside the TLSs at the tumor margin or tumor centre were assessed. Our data showed that the TLS density was significantly positively correlated with CD4^+^ T cells, CD8^+^ T cells, CD20^+^ B cells and CD45RO^+^ memory T cells and negatively correlated with CD15^+^ TANs in the tumor margin. Additionally, the TLS density was significantly positively correlated with CD4^+^ T cells, CD8^+^ T cells, CD20^+^ B cells, CD45RO^+^ memory T cells and NCR1^+^ NK cells and negatively correlated with CD15^+^ TANs in the tumor centre. Together, these results suggest that a high density of TLS might promote an immune‐responsive microenvironment. Recent studies reported that the density of TLSs predicted the therapeutic efficacy of immune checkpoint inhibitors (ICIs); for example, a better efficacy of ICIs corresponded to a high density of TLS and high infiltration of B cells in most tumors, such as melanoma, renal cell carcinoma, small cell lung cancer and urothelial carcinoma.^21^, ^38^, ^39^, ^40^ These findings support the role of TLS in promoting anti‐tumor immunity, consistent with our study of a relatively strong correlation between TLS density and primary tumor‐infiltrating immune cells outside the TLSs.

All the above findings suggest that a high density of TLS might play a promoting role in anti‐tumor immune response and might be a favorable prognostic indicator in GNENs. Since a high density of TLS in GNENs was correlated with good clinical results, we further used the ROC curve to determine the cut‐off value of TLS density; the study cohort was accordingly divided into TLS^High^ and TLS^Low^ groups. Univariate and multivariate survival analyses in the training cohort and external validation set demonstrated that a high TLS density was an independent prognostic indicator of prolonged RFS and OS.

Furthermore, we constructed prognostic nomograms, which accurately predicted the probability of 3‐ and 5‐year OS and RFS of GNEN patients and showed better performance than predictions based on WHO classification, AJCC8 TNM stage or other independent factors. To the best of our knowledge, nomograms consisting of TLS density, AJCC8 TNM stage, WHO classification and tumor size were the first prediction models that contained TLSs for the prognosis of GNENs. The nomograms outperformed the WHO classifications and AJCC TNM staging in terms of discrimination, calibration abilities and clinical utilities. These apply not only to GNET but also to GNEC/GMiNEN. For clinical utilities, when the threshold probability was about 0.5–0.75, the net benefit of the nomogram was higher than the AJCC staging and WHO classifications. This suggests that for some patients with high‐risk resected GNEN, treatment plans based on the nomogram model might achieve higher clinical benefits. These predictive models may be a new visual graph tool for clinicians to estimate the prognosis of patients with resected GNEN and develop individualised patient treatment plans.

Nomogram prediction models for RFS and OS in GNENs have also been previously established in other studies.^8^, ^9^, ^10^ However, these models are subject to certain limitations, primarily relying on clinicopathological characteristics drawn from single‐centre datasets and lacking validation from external cohorts. For instance, Huang *et al*.^10^ proposed a new scoring system with a significantly better prediction ability for RFS in patients with GNEC but could not predict the RFS of patients with GNET. Zhou *et al*.^9^ proposed a nomogram to predict recurrence in GNEN that included tumor T grade, Ki‐67 index and tumor M grade but lacked an external validation set. Hu *et al*.^8^ proposed a nomogram for GNET and GNEC based on the SEER programme and multicentre studies; however, they did not encompass immune‐related factors. Since TIME has been recognised to play an indispensable role in tumor prognosis, we explored the immune aspects of NEC development. Compared with existing models, our models have several advantages. First, our nomograms had larger AUC values compared with any other independent risk factor incorporated into the nomograms and traditional staging systems commonly used. Second, our nomograms were constructed using TLSs – an essential anti‐tumor element in TIME. Third, although our models were constructed based on resected GNEN, they can be applied to GNET and GNEC patients, significantly improving their clinical practicability. Overall, the proposed nomograms have a good prognostic performance.

Nonetheless, this retrospective study has some limitations. First, the cohorts in our study mostly encompassed localised GNENs, with very few metastatic patients; therefore, we only detected prognostic values in resected GNENs. Second, this study did not compare the associations between TLSs and tumor mutational burden (TMB), and immune checkpoint receptors and ligands (such as programmed cell death 1 receptor (PD‐1) and its ligand (PD‐L1/PD‐L2), T cell immunoreceptor with Ig and ITIM domains (TIGIT), CD155, etc.), which will be explored in our future studies. Third, the influence of the predictive value of TLSs in GNEN patients receiving neoadjuvant therapy or immunotherapies should be clarified. Finally, prospective clinical trials and basic research are warranted to determine whether TLS is a promising biomarker in tumor immunotherapies.

In conclusion, high TLS density is an independent favorable prognostic factor for both RFS and OS in resected GNEN patients. Nomograms based on TLS density, tumor size, WHO classification and AJCC8 TNM stage may more rapidly and accurately predict the probability of 3‐ and 5‐year OS and RFS in patients with resected GNEN. The study findings suggest that TLS may play a role in enhancing anti‐tumor immunity, and induction of the formation of TLSs could potentially improve the prognosis of patients with GNEN.

## Methods

### Patient population

Two independent patient cohorts were enrolled in this study. For the training set, 140 patients who underwent surgical or endoscopic resection and had confirmed GNEN by histology at the affiliated Drum Tower Hospital of Nanjing University Medical School from January 2013 to June 2021 were enrolled. Patients who met the following criteria were included in this study: (1) ≥ 18 years of age and ≤ 75 years of age; (2) patients who underwent surgical or endoscopic resection and had confirmed GNEN by postoperative pathology; (3) did not undergo any other treatment before surgery; (4) normal liver and renal function; (5) acceptable cardiovascular pulmonary and other major organ functions; and (6) agreed to undergo follow‐up. Patients who met the following criteria were excluded: (1) patients who received adjuvant therapy (including chemotherapy, radiation, targeted therapy and immuno‐therapy); (2) patients who underwent emergency resection, resection with palliative intent and died within 30 days of surgery; (3) patients who had dual or multiple primary tumors; (4) other serious organ function damage; and (5) follow‐up data were incomplete or missing. Employing similar inclusion and exclusion criteria, an external validation cohort comprising 126 patients with GNEN treated at the Chinese PLA General Hospital between January 2008 and June 2019 was included. Tumor staging was assigned according to the 8th edition of the AJCC gastroenteropancreatic neuroendocrine tumor staging system, encompassing all GNEN cases. In addition, histological grade and classification were evaluated based on the WHO classification 2019. Based on the AJCC TNM stage and WHO classification, the TNM stage is categorised into four stages: I, II, III and IV, and the WHO classification includes neuroendocrine tumor (NET includes G1, G2 and G3), neuroendocrine carcinoma (NEC) and mixed neuroendocrine neoplasm (MiNEN). Considering the unique nature of GNEN, almost all GMiNEN cases have a mixture of GNEC with adenocarcinoma.^41^ Therefore, we assigned GMiNEN and GNEC into one group in this study. Patients were followed up every 3 months in the first 2 years after surgery and then at least once a year thereafter. The study was approved by the Institutional Review Boards of both centres, and informed consent was obtained before surgery for the collection and use of surgical specimens and related clinical data.

### Pathological evaluation

After collection, the tumor samples were formalin fixed, paraffin embedded (FFPE). Initially, all available H&E‐stained sections in the training set were reviewed. Tumor‐related TLS was defined to be within 7 mm of the tumor border, including the tumor region based in line with previous findings.^31^, ^37^ We only included specimens containing both tumor tissue and surrounding para‐tumor tissue in this study. Specimens with tumor boundaries less than 7 mm were excluded to ensure accurate TLS counts. The corresponding FFPE tissue blocks were sectioned at 4 μm for staining. Subsequently, H&E‐stained sections from the 126‐patient external validation set were obtained from the Chinese PLA General Hospital, following selection using the same criteria. To further evaluate the cellular composition of TLS in GNEN, IHC staining was conducted using 140 serial sections from the training cohort as previously described.^34^ IHC staining was performed to detect helper T cells (Abcam, ab133616, rabbit monoclonal to CD4, 1:200, Cambridge, UK), cytotoxic T cells (Abcam, ab178089, rabbit monoclonal to CD8 alpha, 1:100), regulatory T cells (Abcam, ab20034, mouse monoclonal to FOXP3, 1:500), memory T cells (Abcam, mouse monoclonal to CD45RO, 1:1000), B cells (Abcam, ab78237, rabbit monoclonal to CD20, 1:100), dendritic cells (Abcam, ab52632, rabbit monoclonal to CD11c, 1:500), natural killer cells (Abcam, ab224703, rabbit monoclonal to NCR1, 1:1000) and tumor‐associated neutrophils (Santa Cruz, SC‐21702, mouse monoclonal to CD15, 1:100, CA, USA). After the staining procedure, the pathological sections from each patient were scanned for WSIs, which comprised tissues from both the tumor and adjacent surrounding normal tissues. Scanning control software (K‐Scanner 1.6.0.14, Konfoong Biotech International Co. Ltd, Ningbo, China) and image browsing and management software (K‐Viewer 1.5.3.1, Konfoong Biotech International Co. Ltd) were utilised for control scanning and viewing in WSIs.^42^

To examine the localisation and abundance of multiple immune cells of TLS in the same slide of GNEN tissue, fluorescent mIHC^13^, ^43^, ^44^ was performed on serial sections of FFPE tumor tissue using a Six Colour mIHC Fluorescence kit (Recordbio Biological Technology, Shanghai, China) based on the tyramide signal amplification (TSA) technology following the manufacturer's instructions. Immune cells were stained with CD4^+^ T cells, CD8^+^ T cells, CD20^+^ B cells, CD15^+^ TANs and FOXP3^+^ Treg antibodies. Cell nuclei were stained with DAPI (4′,6‐diamidino‐2‐phenylindole, a blue‐emitting fluorescent compound used for nuclear staining). Multiplexed colour slides were scanned with a PerkinElmer Vectra automated multispectral microscope at 600× magnification (Vectra 3; PerkinElmer, Hopkinton, MA) and quantitative image analysis was performed using the HALO V.3.2 image analysis platform (Indica Labs, Albuquerque, New Mexico, USA).

Tertiary lymphoid structures are organised aggregates of immune cells which are formed in non‐lymphoid tissues at sites of chronic inflammation, such as in autoimmune disease, chronic infection and cancer. They exhibit a pronounced anatomical and functional resemblance to secondary lymphoid organs (lymph nodes and Peyer's patches) but lack the surrounding capsule.^15^ Their morphology can be detected on WSIs by trained observers, and all WSIs were reviewed by two independent observers to determine the number, location and maturity classification of TLS (they were fully blinded to the clinical characteristics of the patients). Tumor‐related TLS was defined to be within 7 mm of the tumor border, including the tumor region.^37^ The area of tumor‐related TLS was calculated using ImageJ software (NIH Image J System, Bethesda, MD, USA).^45^ TLS density was calculated as the number of TLS per mm^2^ tumor‐related TLS regions in the WSIs. The maturity and identity of TLS were evaluated as previously described.^36^ The numbers of peritumoral and intra‐tumoral infiltrating immune cells, which did not belong to the TLS, were estimated in five random high‐power fields (HPF; ×200). Cell counting was conducted in corresponding regions using Image J.^46^ Data are expressed as the mean ± standard error of the mean (sem). The percentage of each cell component in the TLS was calculated as the number of cells of each type in all nucleated cells in the TLS. Next, the average value of each cell component of TLS in the whole slide was calculated as the representative value of a patient.

### Statistical analysis

Statistical analyses were performed using the R Studio (version 3.6.3) and SPSS (version 26.0). Categorical variables were compared with the χ^2^ test or Fisher's exact test, while the *t‐*test, analysis of variance or Mann–Whitney *U*‐test was used to compare continuous variables. Receiver operating characteristic curves were constructed to measure the cut‐off values for discriminating between patients with or without death. Kaplan–Meier curves of RFS and OS were plotted for TLS density. The significance of each predictor variable was initially assessed using univariate Cox regression analysis. Variables found to be statistically significant (*P* < 0.05) were then included in a separate multivariate Cox regression analysis to identify independent prognostic factors. Nomograms for predicting OS and RFS were constructed using the R library ‘rms’ package. The Nomogram was first internally validated using the bootstrap method and then externally validated in independent cohorts. The concordance index (C‐index) values were calculated and employed to evaluate the predictive performance of each factor, with 0.5 indicating random chance, and closer to 1.0 indicating a better ability to correctly discriminate the outcome. Calibration curves^47^ and DCA^48^, ^49^, ^50^ were employed to assess the performance of the developed nomograms. The ‘Calibration Curves’ package was used to create calibration curves. The ‘Decision Curve’ package was utilised to perform DCA. In addition, time‐dependent ROC curves of the nomograms and all independent prognostic variables at 3 and 5 years were generated, and the corresponding AUC of time‐dependent ROC was used to evaluate discrimination.

## Author contributions

**Daming Cai:** Conceptualization; data curation; formal analysis; methodology; software; validation; visualization; writing – original draft; writing – review and editing. **Xingzhou Wang:** Conceptualization; data curation; investigation; methodology; software; writing – review and editing. **Heng Yu:** Conceptualization; investigation; methodology; writing – review and editing. **Chunhua Bai:** Data curation; investigation; methodology. **Yonghuan Mao:** Data curation; methodology. **Mengjie Liang:** Data curation; validation. **Xuefeng Xia:** Investigation; methodology; supervision. **Song Liu:** Methodology; supervision. **Meng Wang:** Methodology; supervision. **Xiaofeng Lu:** Methodology; supervision. **Junfeng Du:** Methodology; project administration; supervision. **Xiaofei Shen:** Conceptualization; funding acquisition; methodology; project administration; supervision; writing – review and editing. **Wenxian Guan:** Funding acquisition; project administration; supervision; writing – review and editing.

## Conflict of interest

The authors declare no conflict of interest.

## Ethics approval

This study was approved by the institutional research ethics committee of the affiliated Drum Tower Hospital of Nanjing University Medical School (2017–235‐01) and Chinese PLA General Hospital (S2017‐134‐01). According to the ethics committee regulations, informed consent was obtained from patients enrolled in this study.

## Supporting information


Supplementary figures 1‐12

## Data Availability

All data generated in this study are included in the article or uploaded as Supporting Information. Additional datasets can be requested from the corresponding authors.
