# Supplementary material for: Infiltrating characteristics and prognostic value of tertiary lymphoid structures in resected gastric neuroendocrine neoplasm patients
Source: Clin Transl Immunology. 2024 Feb 5;13(2):e1489. doi: 10.1002/cti2.1489 (PMC10844765; doi:10.1002/cti2.1489)
Supplement: Supplementary file 1 — Supplementary figures 1‐12 [file CTI2-13-e1489-s001.pdf]

Supplementary figure 1

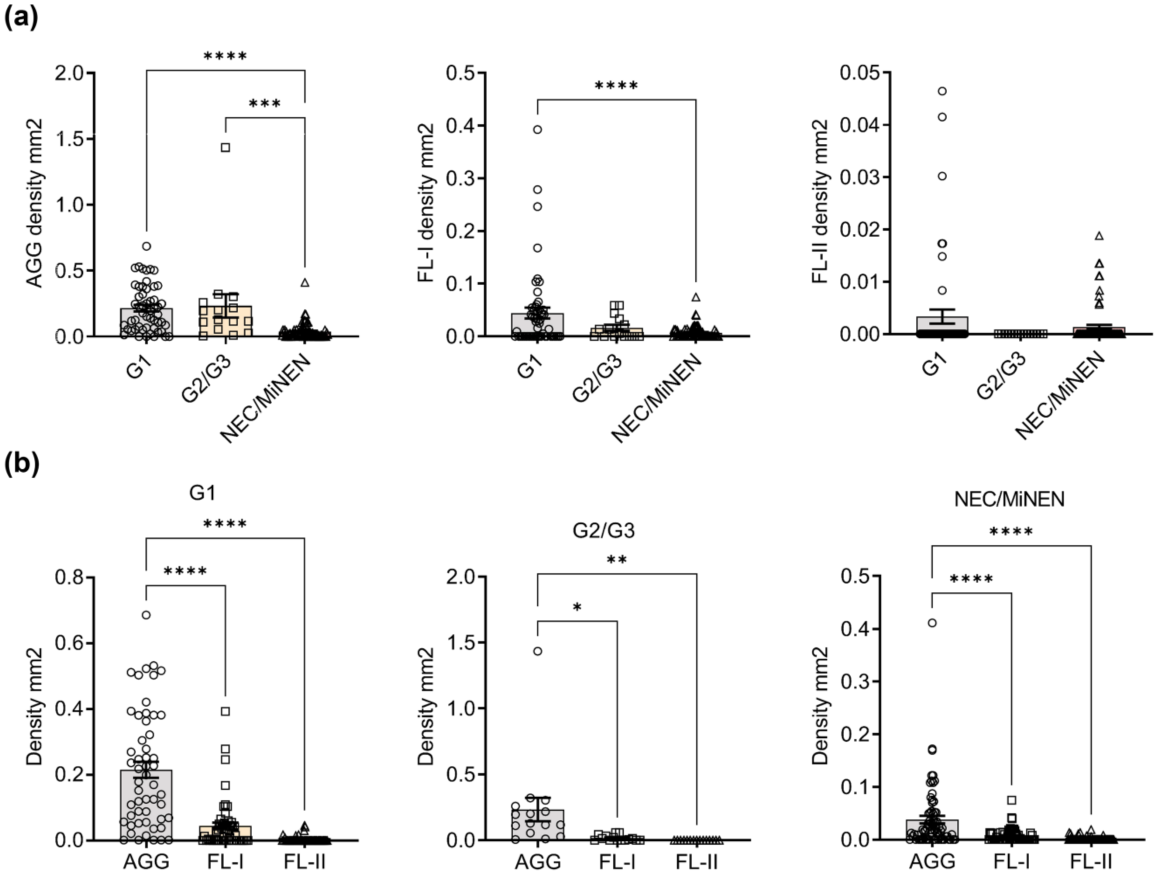

Supplementary figure 1. Relationship between the density of TLS with different maturity classification and WHO classification (**a, b**). (NS means no significance, * means $P < 0.05$, ** means $P < 0.01$ and *** means $P < 0.001$) (The maturity classification of TLS was described in the main text, containing Aggregates (AGG), Primary mature TLS(FL-I), and Secondary mature TLS(FL-II)).

Supplementary figure 2

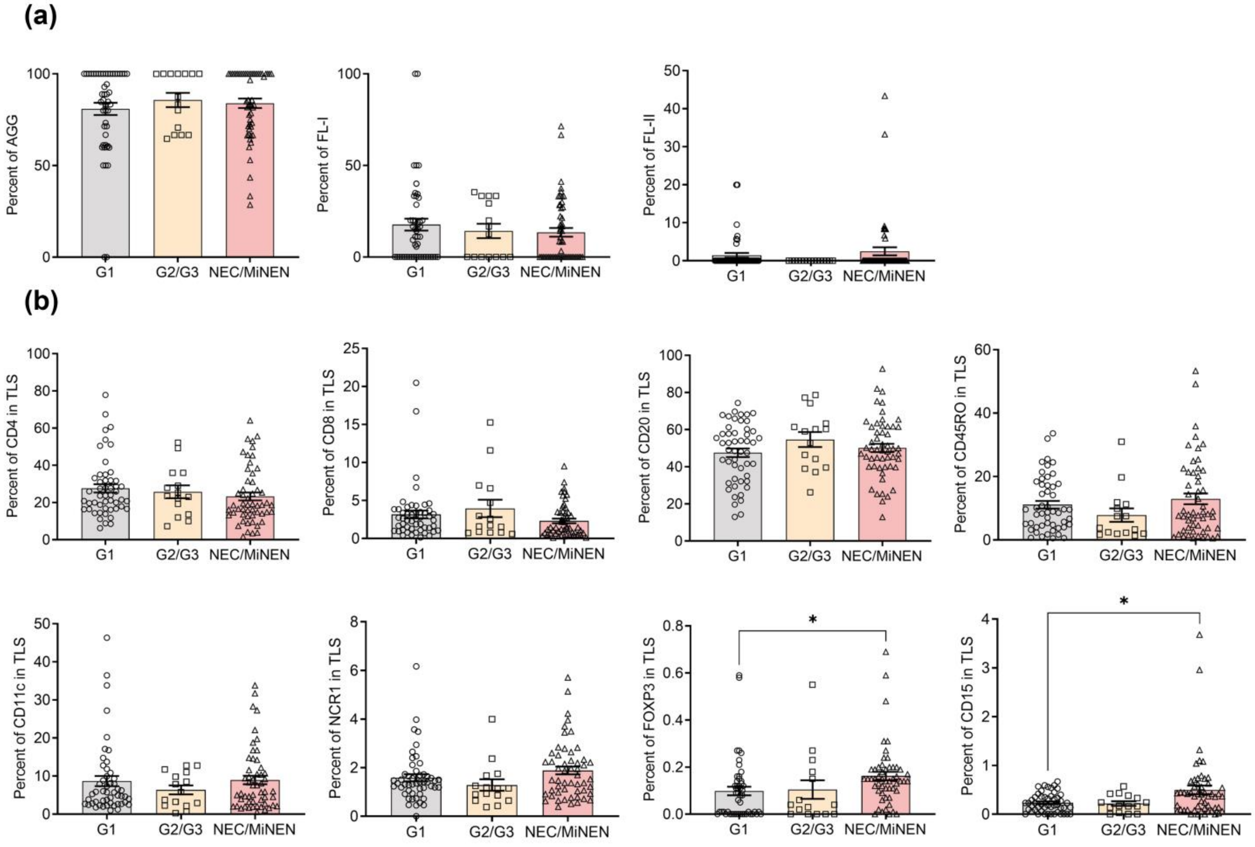

Supplementary figure 2. The distribution of TLS maturity and cell components of TLS with different WHO classification (**a, b**). (* means *P* < 0.05).

Supplementary figure 3

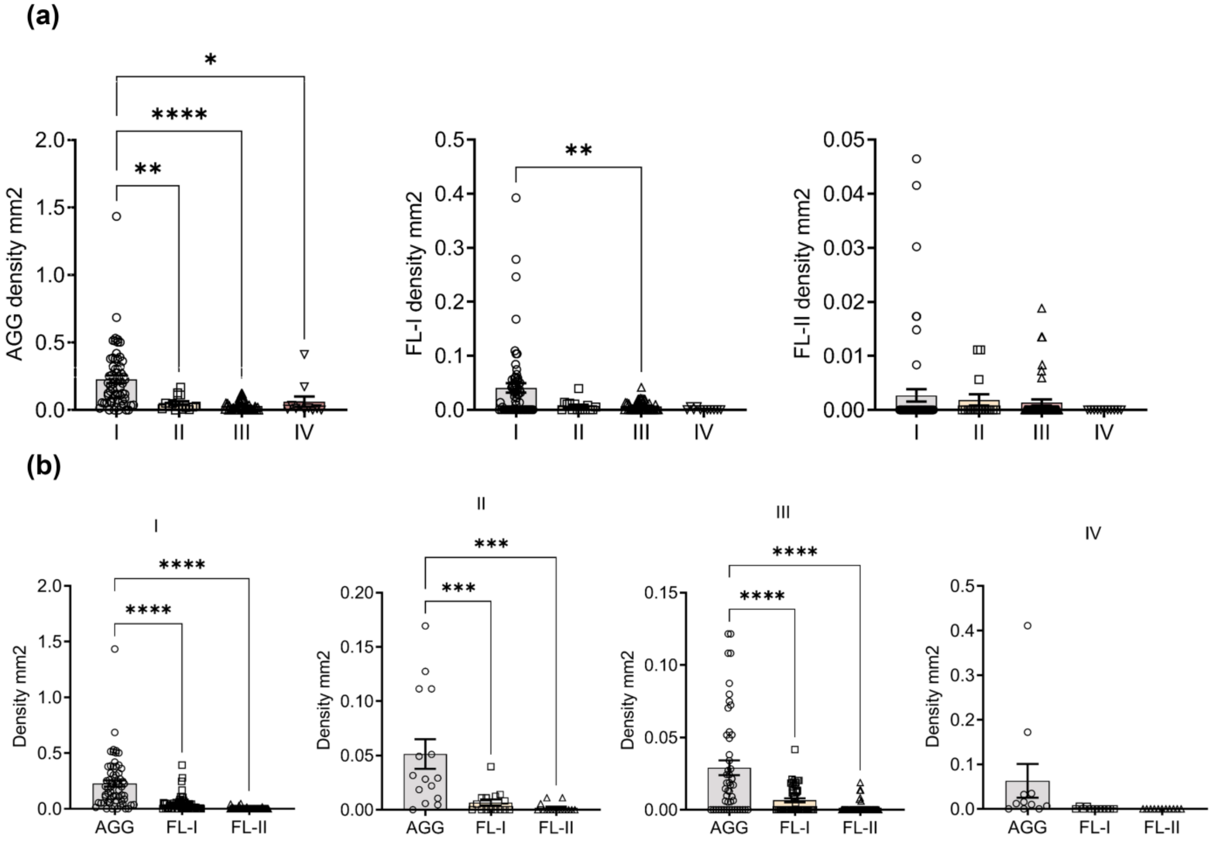

Supplementary figure 3. Relationship between the density of TLS with different maturity and AJCC8thTNM stage (**a, b**). (* means $P < 0.05$, ** means $P < 0.01$, *** means $P < 0.001$ and **** means $P < 0.0001$) (The maturity classification of TLS was described in the main text, containing Aggregates (AGG), Primary mature TLS(FL-I), and Secondary mature TLS(FL-II)).

Supplementary figure 4

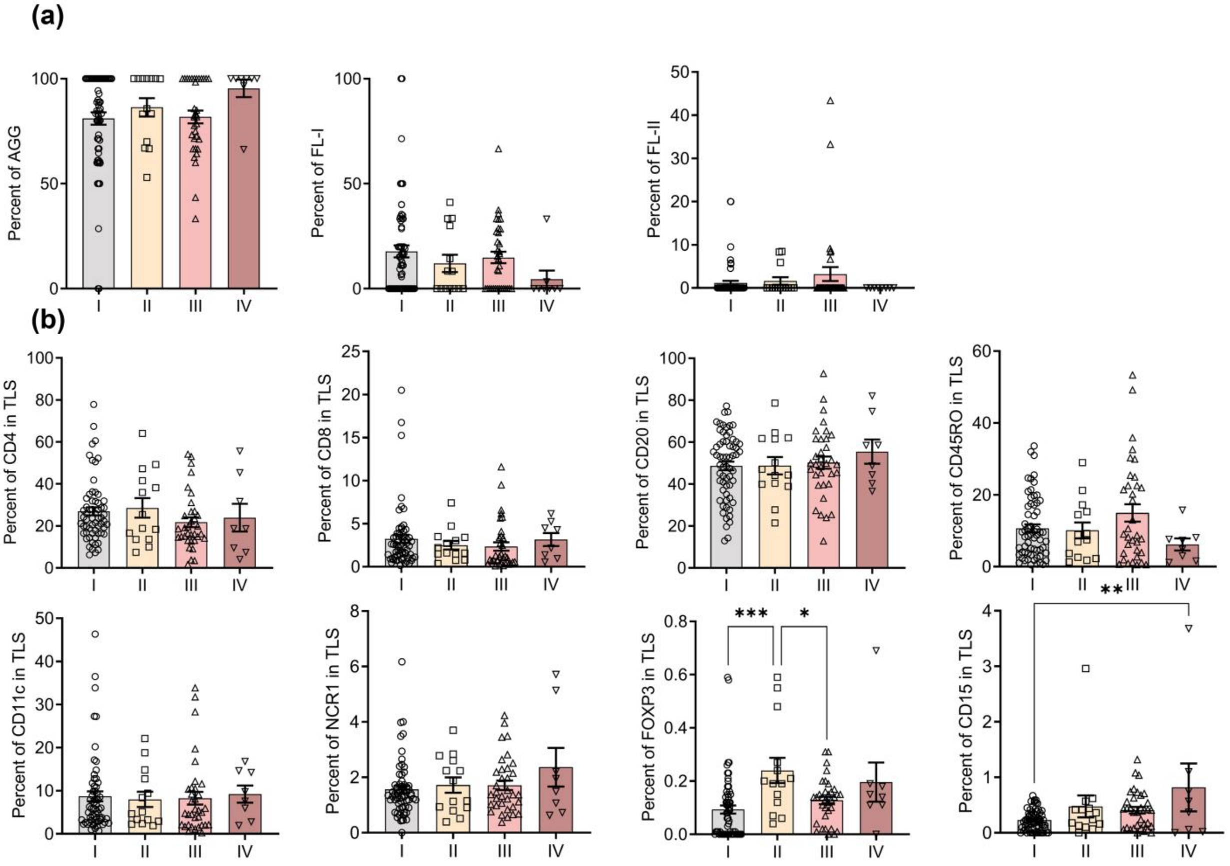

Supplementary figure 4. The distribution of TLS maturity and cell components of TLS with different AJCC8thTNM stage (**a, b**). (* means *P* < 0.05, ** means *P* < 0.01, and *** means *P* < 0.001).

Supplementary figure 5

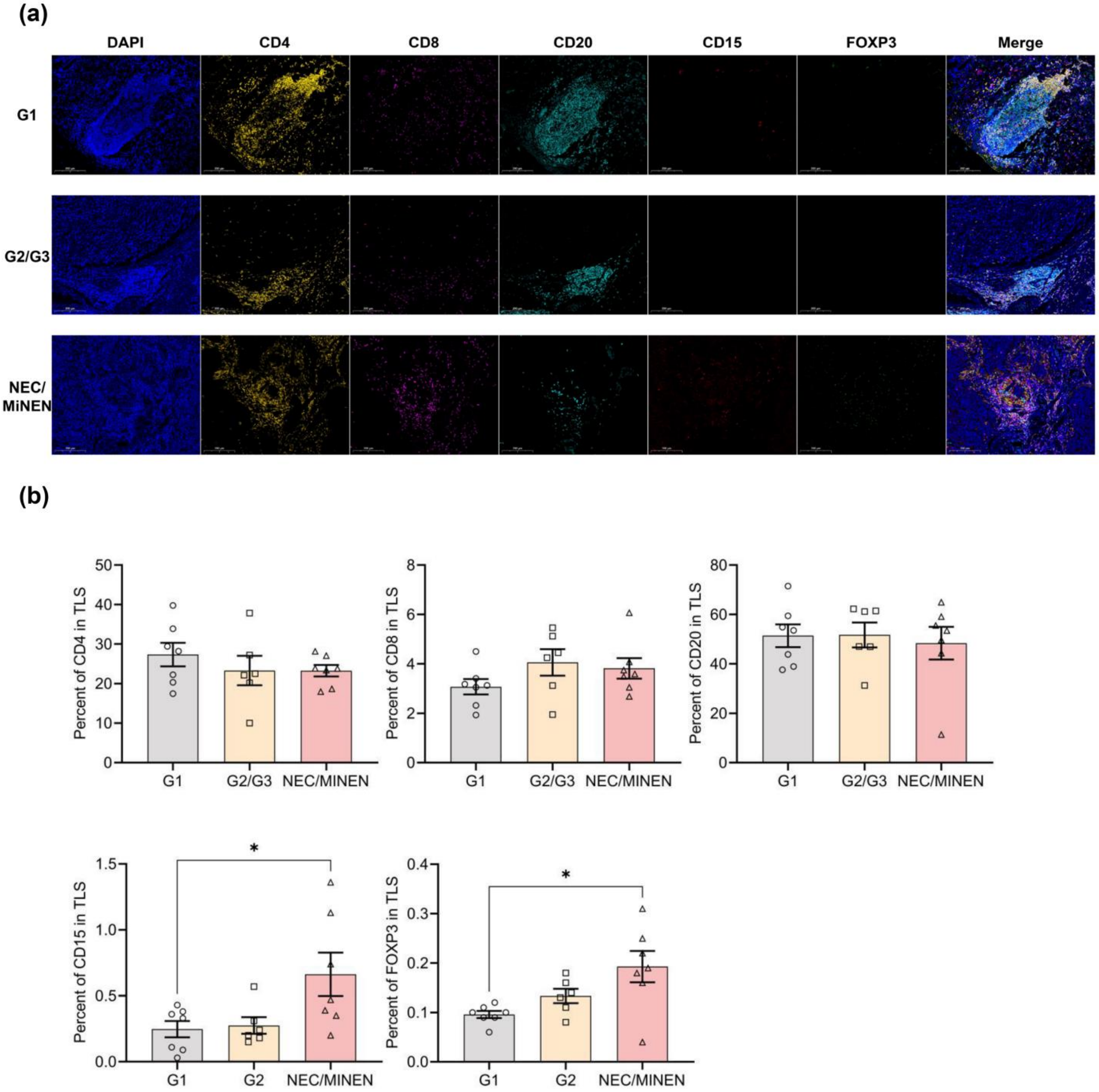

Supplementary figure 5. (**a**) Multispectral fluorescent immunohistochemistry on same slide of GNEN. In the first row, representative immunofluorescence images of TLS in tissue of G1 GNET. In the second row, representative immunofluorescence images of TLS in tissue of G2/G3 GNET. In the third row, representative immunofluorescence images of TLS in tissue of GNEC/MiNEN. Magnification: 200×. Scale bars correspond to 200 µm. (**b**)The cell components of TLS with different WHO classification in mIHC slides. (* means $P < 0.05$).

Supplementary figure 6

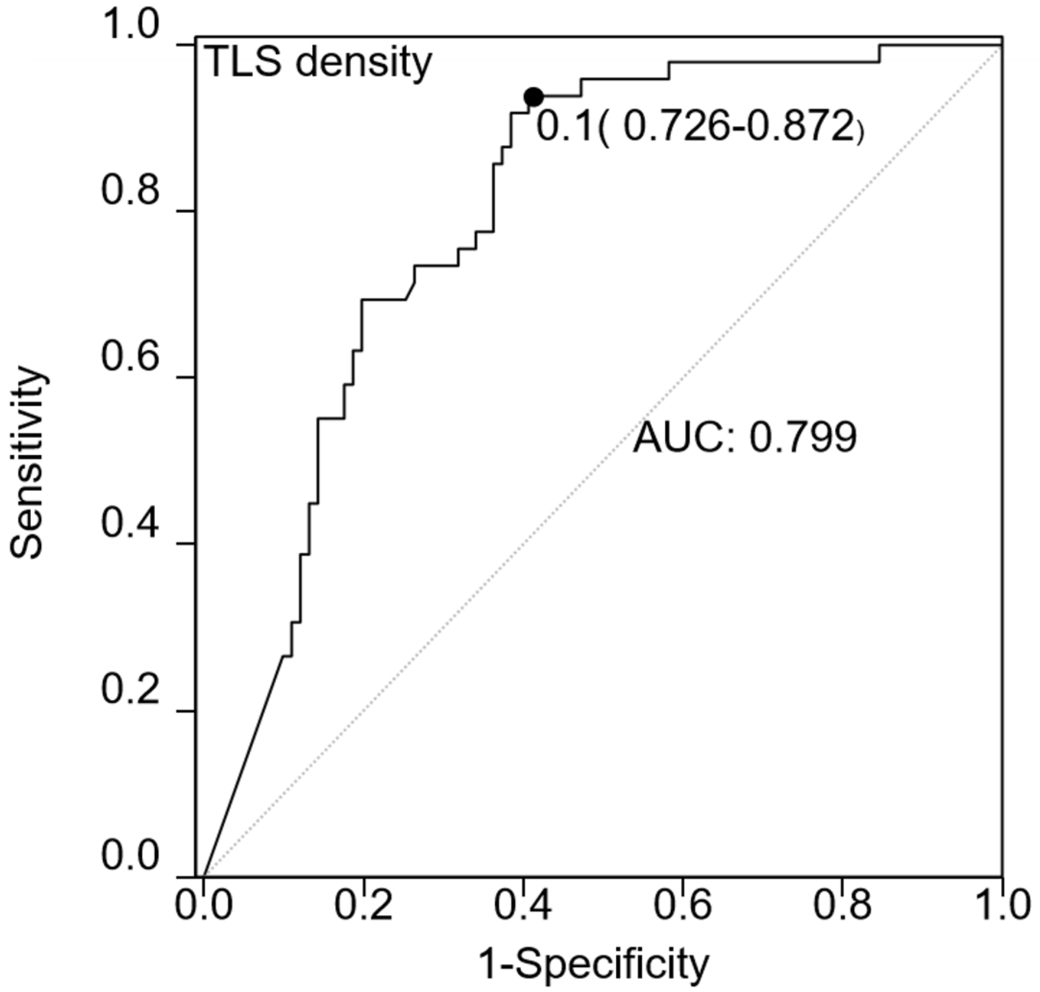

Supplementary figure 6. A receiver operating characteristic (ROC) curve was used to determine cut-off values for TLS density. The optimum cut-off is 0.1 per mm2, and when using this point for segmentation, the sensitivity is 0.918, and the specificity is 0.615. The area of tumor-related TLS was calculated by Image J, and the TLS density was calculated as the number of TLSs per mm2 of tumor-related TLS region in WSIs. The cut-off point of TLS density was determined using the receiver operating characteristics (ROC) curve analysis. The ROC curve was constructed using survival status and TLS density; the best cut-off was when the AUC was the maximum (The maximum of the AUC is 0.799, 95%CI is 0.726 ~ 0.872).

Supplementary figure 7

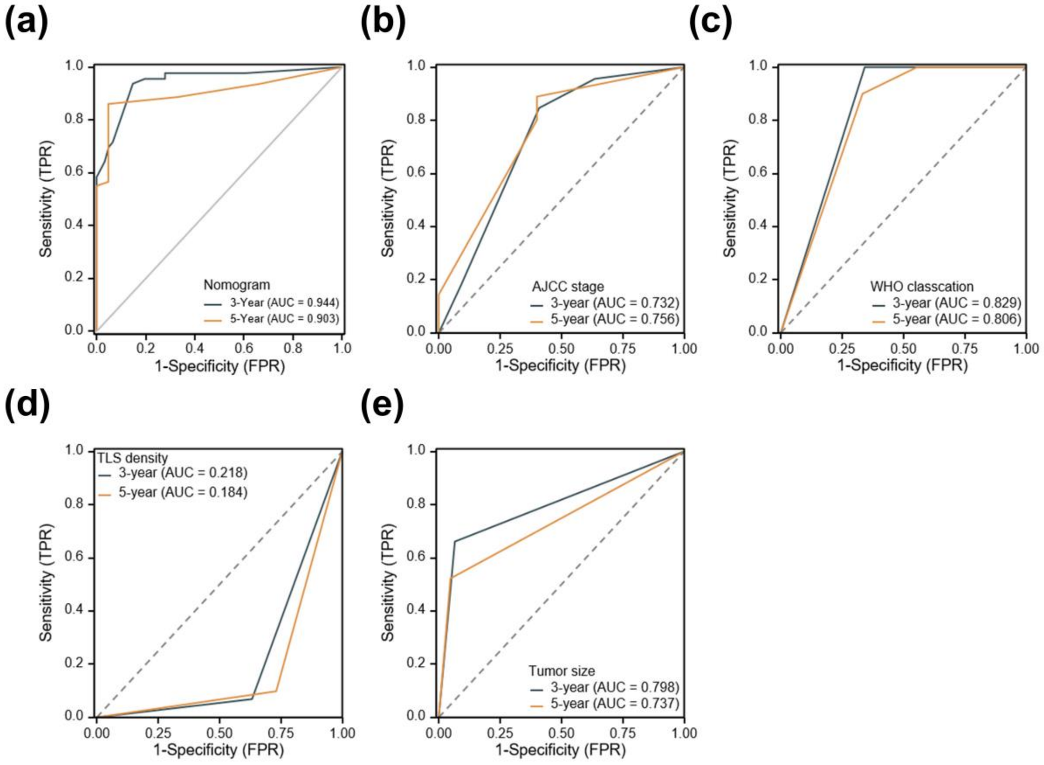

Supplementary figure 7. Time-dependent ROC analysis of the Nomogram, AJCC stage, WHO classification, TLS density, and tumor size for 3 and 5-year OS of patients with GNEN in the training set. (**a**) ROC curve of the developed prediction model. (**b**) ROC curve of the AJCC stage. (**c**) ROC curve of WHO classification. (**d**) ROC curve of TLS density. (**e**) ROC curve of tumor size.

Supplementary figure 8

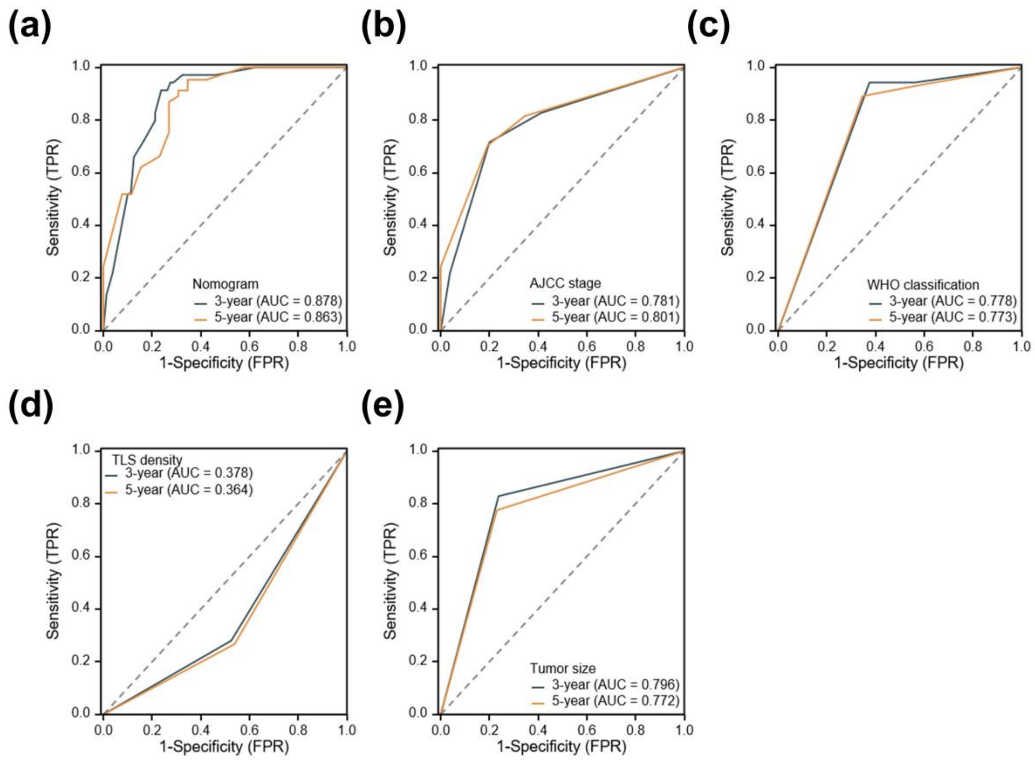

Supplementary figure 8. Time-dependent ROC analysis of the Nomogram, AJCC stage, WHO classification, TLS density, and tumor size for 3 and 5-year OS of patients with GNEN in the external validation set. (**a**) ROC curve of the developed prediction model. (**b**) ROC curve of the AJCC stage. (**c**) ROC curve of WHO classification. (**d**) ROC curve of TLS density. (**e**) ROC curve of tumor size.

Supplementary figure 9

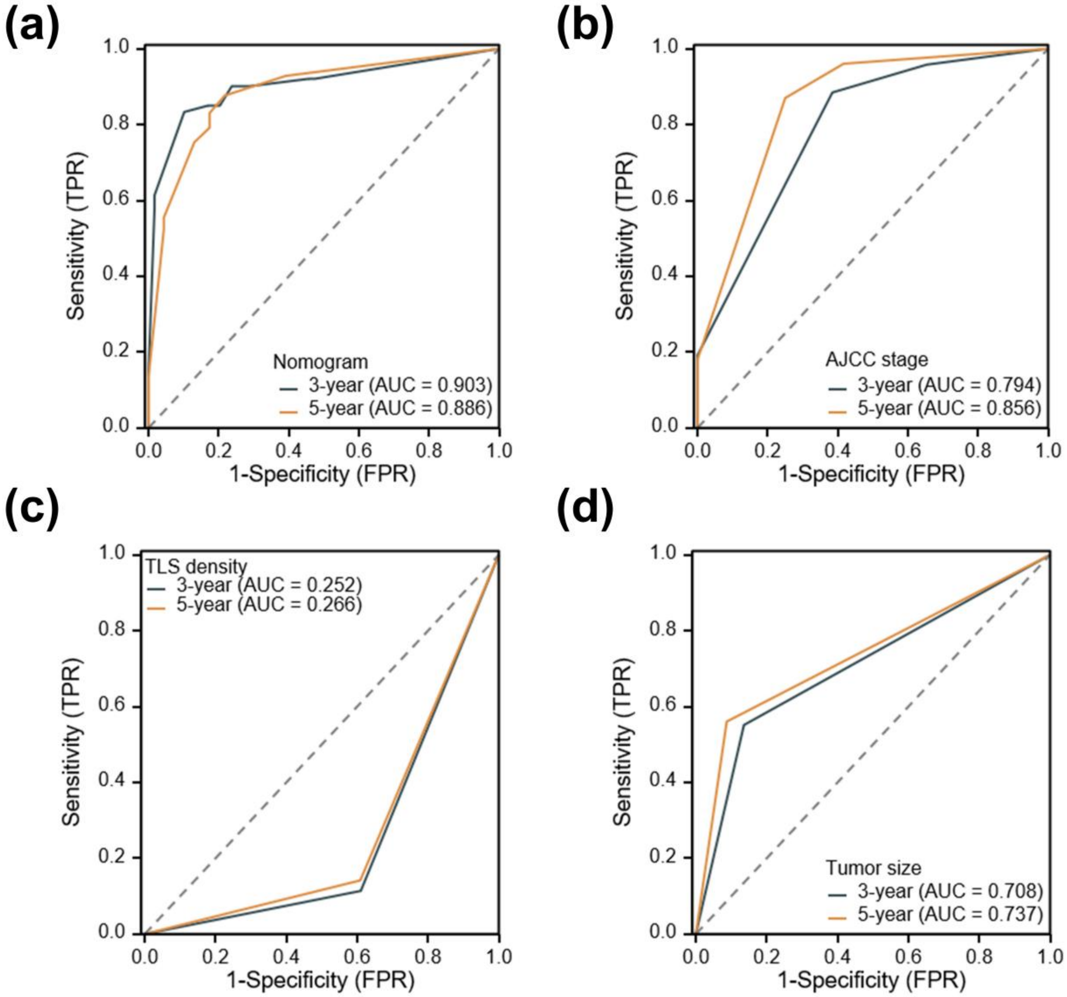

Supplementary figure 9. Time-dependent ROC analysis of the nomogram, AJCC stage, TLS density, and tumor size for 3 and 5-year RFS for the patients with GNEN in the training set. (**a**) ROC curve of the developed prediction model. (**b**) ROC curve of the AJCC stage. (**c**) ROC curve of TLS density. (**d**) ROC curve of tumor size.

Supplementary figure 10

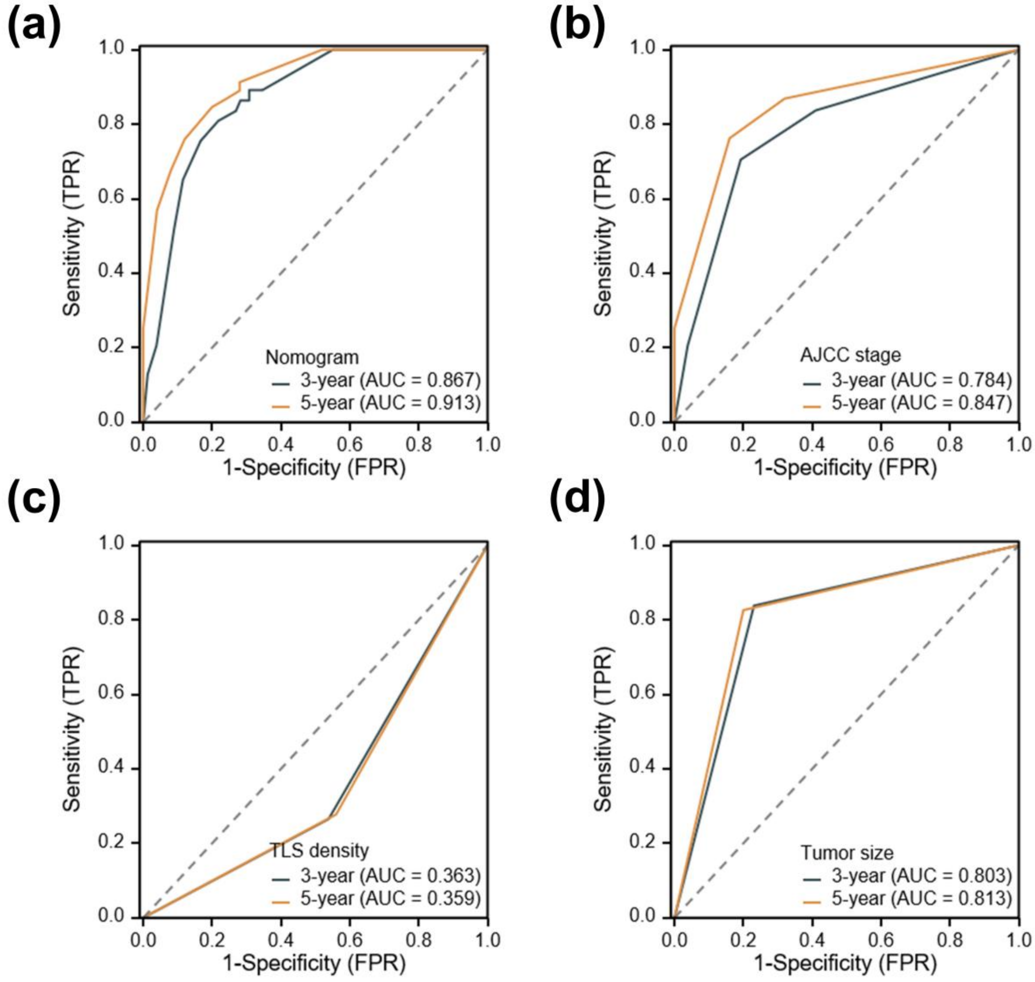

Supplementary figure 10. Time-dependent ROC analysis of the nomogram, AJCC stage, TLS density, and tumor size for 3 and 5-year RFS for the patients with GNEN in the external validation set. (**a**) ROC curve of the developed prediction model. (**b**) ROC curve of the AJCC stage. (**c**) ROC curve of TLS density. (**d**) ROC curve of tumor size.

Supplementary figure 11

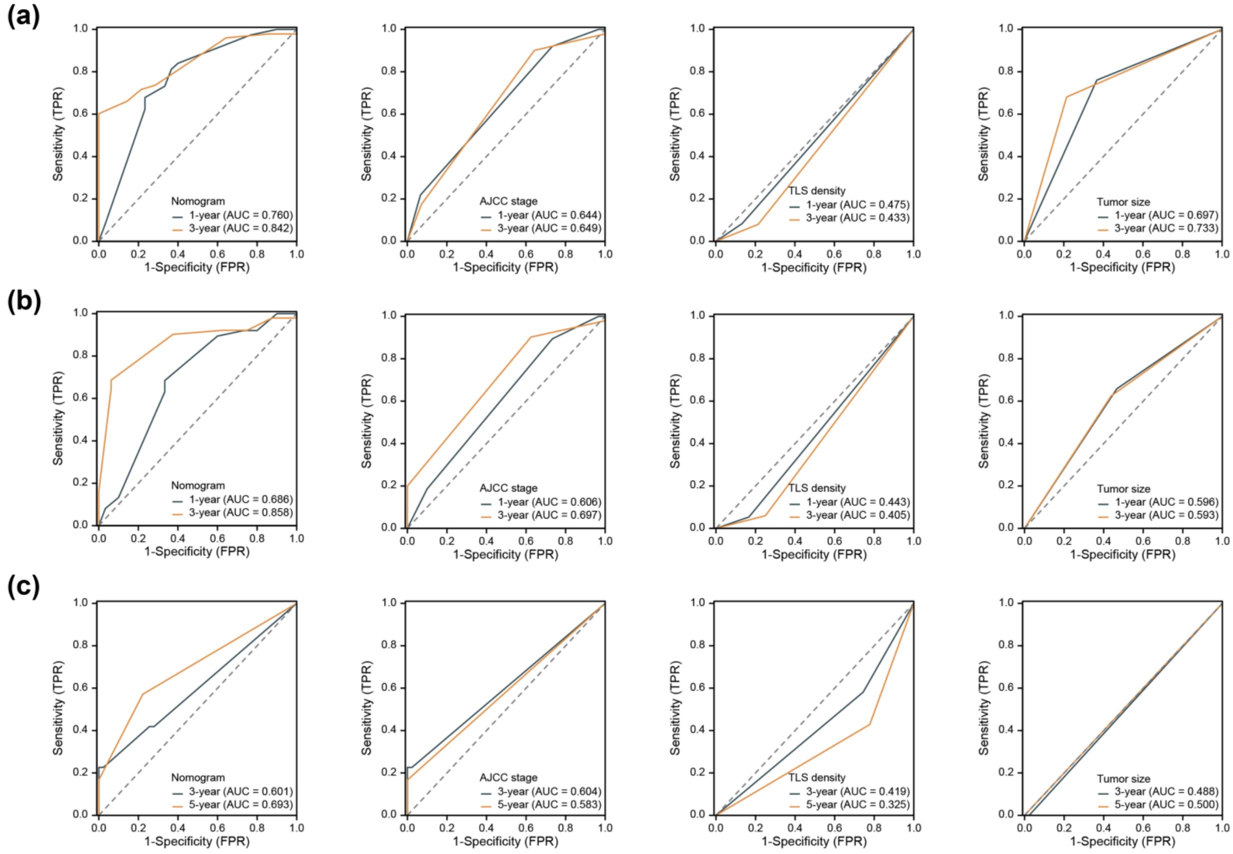

Supplementary figure 11. Time-dependent ROC analysis of the nomograms, AJCC stage, TLS density, and tumor size in the training set. **(a)** 1 and 3-year OS for the patients with GNEC **(b)** 1 and 3-year RFS for the patients with GNEC **(c)** 3 and 5-year RFS for the patients with GNET.

Supplementary figure 12

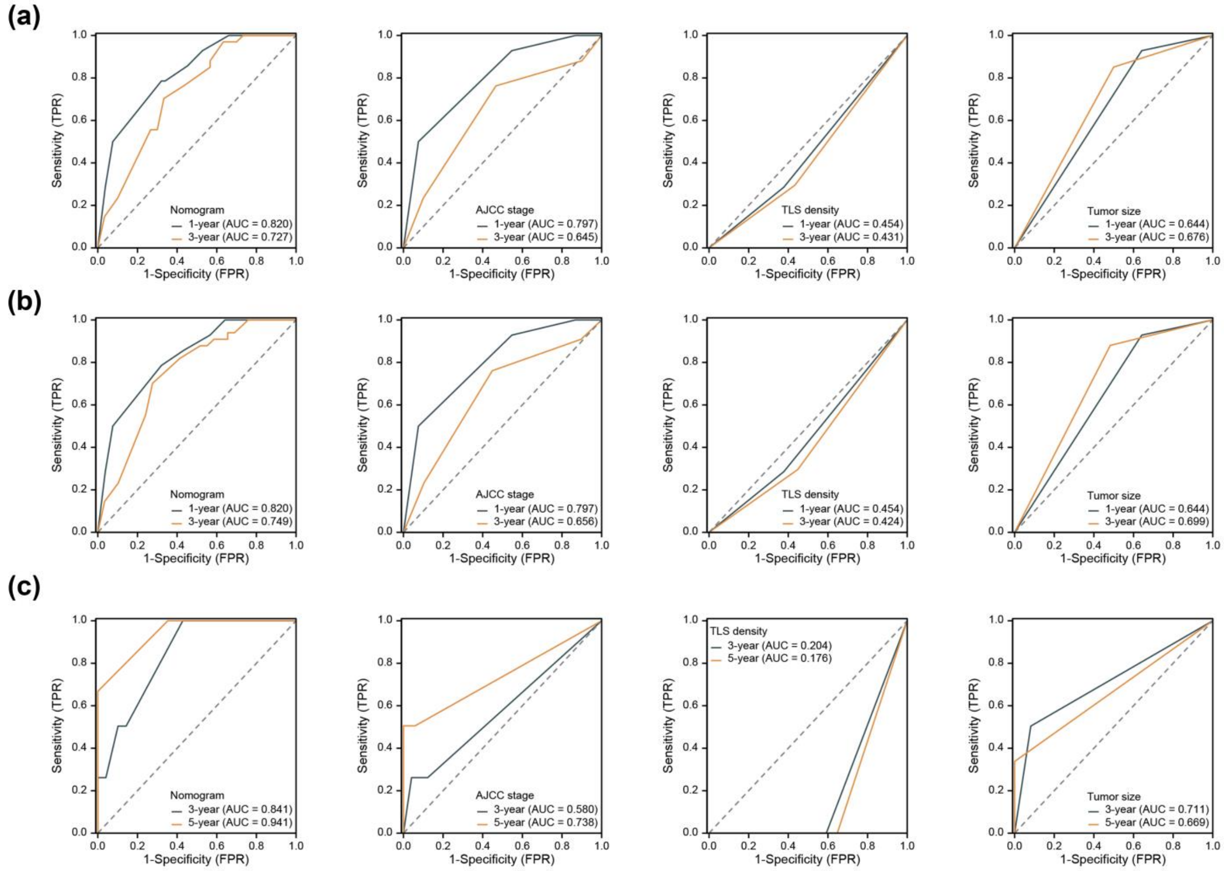

Supplementary figure 12. Time-dependent ROC analysis of the nomograms, AJCC stage, TLS density, and tumor size in the external validation set. **(a)** 1 and 3-year OS for the patients with GNEC **(b)** 1 and 3-year RFS for the patients with GNEC **(c)** 3 and 5-year RFS for the patients with GNET.
